# Identification and characterization of two functional variants in the human longevity gene *FOXO3*

**DOI:** 10.1038/s41467-017-02183-y

**Published:** 2017-12-12

**Authors:** Friederike Flachsbart, Janina Dose, Liljana Gentschew, Claudia Geismann, Amke Caliebe, Carolin Knecht, Marianne Nygaard, Nandini Badarinarayan, Abdou ElSharawy, Sandra May, Anne Luzius, Guillermo G. Torres, Marlene Jentzsch, Michael Forster, Robert Häsler, Kathrin Pallauf, Wolfgang Lieb, Céline Derbois, Pilar Galan, Dmitriy Drichel, Alexander Arlt, Andreas Till, Ben Krause-Kyora, Gerald Rimbach, Hélène Blanché, Jean-François Deleuze, Lene Christiansen, Kaare Christensen, Michael Nothnagel, Philip Rosenstiel, Stefan Schreiber, Andre Franke, Susanne Sebens, Almut Nebel

**Affiliations:** 1 Institute of Clinical Molecular Biology, Kiel University, University Hospital Schleswig-Holstein, Campus Kiel, Rosalind-Franklin-Straße 12, 24105 Kiel, Germany; 2 Department of Internal Medicine I, University Hospital Schleswig-Holstein, Campus Kiel, Arnold-Heller-Straße 3, 24105 Kiel, Germany; 3 Institute of Medical Informatics and Statistics, Kiel University, University Hospital Schleswig-Holstein, Campus Kiel, Brunswiker Straße 10, 24105 Kiel, Germany; 4 The Danish Aging Research Center, and the Danish Twin Registry, Epidemiology, Biostatistics and Biodemography, Department of Public Health, University of Southern Denmark, J. B. Winslows Vej 9B, 5000 Odense C, Denmark; 5 Faculty of Sciences, Division of Biochemistry, Chemistry Department, Damietta University, 34511 New Damietta City, Egypt; 6 Institute of Human Nutrition and Food Science, Kiel University, Hermann-Rodewald-Straße 6, 24118 Kiel, Germany; 7 Institute of Epidemiology, Kiel University, University Hospital Schleswig-Holstein, Campus Kiel, Niemannsweg 11, 24105 Kiel, Germany; 8 Centre National de Recherche en Génomique Humaine CNRGH-CEA, 91000 Evry, France; 9 Université Sorbonne Paris Cité-UREN, Unité de Recherche en Epidémiologie Nutritionnelle, U557 Inserm, U1125 Inra, Cnam, Université Paris 13, CRNH IdF, 93000 Bobigny, France; 10 Department of Statistical Genetics and Bioinformatics, Cologne Center for Genomics, University of Cologne, Weyertal 115b, 50931 Cologne, Germany; 11 Institute of Reconstructive Neurobiology and Life & Brain GmbH, University of Bonn, Sigmund-Freud-Straße 25, 53127 Bonn, Germany; 12 Max Planck Institute for the Science of Human History, Kahlaische Straße 10, 07745 Jena, Germany; 13 Fondation Jean Dausset-Centre d’Etude du Polymorphisme Humain (CEPH), 27 Rue Juliette Dodu, 75010 Paris, France; 14 Department of Clinical Genetics, and Department of Clinical Biochemistry and Pharmacology, Odense University Hospital, Sdr. Boulevard 29, 5000 Odense C, Denmark; 15 Institute for Experimental Cancer Research, Kiel University, University Hospital Schleswig-Holstein, Campus Kiel, Arnold-Heller-Straße 3, 24105 Kiel, Germany

## Abstract

*FOXO3* is consistently annotated as a human longevity gene. However, functional variants and underlying mechanisms for the association remain unknown. Here, we perform resequencing of the *FOXO3* locus and single-nucleotide variant (SNV) genotyping in three European populations. We find two *FOXO3* SNVs, rs12206094 and rs4946935, to be most significantly associated with longevity and further characterize them functionally. We experimentally validate the in silico predicted allele-dependent binding of transcription factors (CTCF, SRF) to the SNVs. Specifically, in luciferase reporter assays, the longevity alleles of both variants show considerable enhancer activities that are reversed by IGF-1 treatment. An eQTL database search reveals that the alleles are also associated with higher *FOXO3* mRNA expression in various human tissues, which is in line with observations in long-lived model organisms. In summary, we present experimental evidence for a functional link between common intronic variants in *FOXO3* and human longevity.

## Abstract (web summary)

*FOXO3* is one of the few established longevity genes. Here, the authors fine-map the *FOXO3*-longevity association to two intronic SNPs and, using luciferase assays and EMSAs, show that these SNPs affect binding of transcription factors CTCF and SRF and associate with *FOXO3* expression.

## Introduction

Human longevity is a complex phenotype with modest heritability^1^. The identification of the involved genes and variants still remains a challenge even though the first ever genetic association of the apolipoprotein E allele ε4 with longevity was already reported in 1994^2^. Since then, this finding has been replicated in numerous studies^3,4^. Variation in the forkhead box O3 (*FOXO3*) gene is the only other consistent factor that was shown to influence longevity across diverse populations, including Japanese-Americans^5^, Germans^6^, and Danes^7^. Interestingly, the effect of the *FOXO3* association is very strong in long-lived individuals (LLI) beyond 95 years of age, and especially in centenarians^6^. Although *FOXO3* is consistently annotated as a human longevity gene, the functional variants and the underlying mechanisms have not been identified yet. The large majority of associated single-nucleotide variants (SNVs) are located in the 3′-UTR of the gene and cluster in or near the last intron^6^. Preliminary sequence analyses indicate that *FOXO3* coding variants are unlikely to play a role in longevity^8–10^. This observation is supported by the fact that the transcription factor FOXO3 is evolutionarily highly conserved^11^. FOXO3 regulates the insulin receptor/insulin-like growth factor-I signaling (IIS) pathway^12^ and is, amongst others, involved in energy metabolism, oxidative stress, apoptosis, cell-cycle regulation and stem cell homeostasis (reviewed in ref. ^13^). Genetic manipulations of *FOXO3* orthologs lead to lifespan extension in various model organisms, mainly via an increased expression of the gene (reviewed in refs ^14,15^). Enhanced *FOXO3* transcription may also mediate a similar beneficial effect in humans^13,16^. In addition, *FOXO3* activity or expression can be modulated by dietary interventions like dietary restriction^17,18^ and phytochemicals such as epigallocatechin gallate (EGCG) in green tea^19^.

The aim of the present study was to identify and characterize functional variants in the *FOXO3* gene. We report on the characterization of the two associated SNVs rs12206094 and rs4946935 that were detected after resequencing of the *FOXO3* gene and association testing in German, French, and Danish longevity samples. We validate in silico predicted SNV allele-dependent binding of transcription factors (CTCF, SRF) experimentally. Luciferase reporter assays show enhancer activities for the longevity alleles of both SNVs, which are reversed by insulin-like growth factor 1 (IGF-1) treatment. An expression quantitative trait loci (eQTL) database search reveals SNV allele-specific associations with *FOXO3* mRNA expression. Overall, our data provide evidence for a functional link between common intronic variants in *FOXO3* and the longevity phenotype in humans.

## Results

### Resequencing and fine-mapping of the *FOXO3* gene

For the detection of common and rare variants, the whole *FOXO3* gene region was resequenced using two next-generation sequencing (NGS) technologies—sequencing by ligation (SBL, SOLiD) and sequencing by synthesis (SBS, Illumina)—and the Sanger method in a total of 118 German LLI (99–110 years), 152 younger German controls (60–75 years) and six HapMap-CEU individuals. The samples used for the different sequencing technologies are provided in Supplementary Table 1. SBL and SBS resulted in a total of 1,081 unique SNV calls with an overlap of 293 SNVs (Supplementary Figs. 1 and 2). Sanger sequencing of the promoter, the exons and exon-intron boundaries led to the detection of 42 SNVs. As 17 of these SNVs had already been observed by NGS, the total number of variants amounted to 1,106 (Supplementary Data 1, Supplementary Fig. 2).

**Fig. 1:**
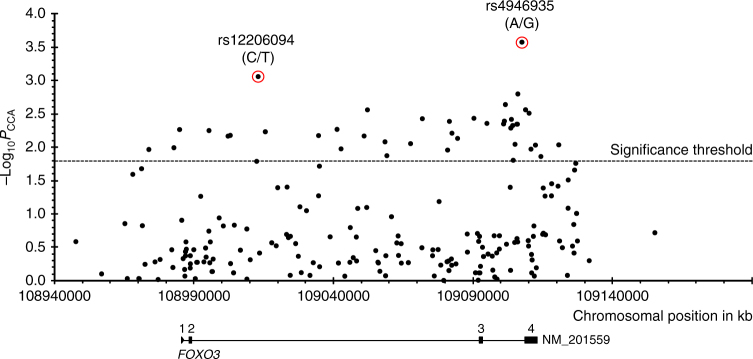
Association plot shows all 205 SNVs tested in the *FOXO3* gene region on chromosome 6 (hg18). The two SNVs with the best *P* values are indicated (red circles). The four exons are shown in the gene model. Number of individuals: 594 German long-lived individuals (≥100 years) and 918 controls (60–75 years). *P*_CCA_, allelic *P* values, determined in a case–control association study by chi-squared test with one degree of freedom; −log_10_*P*_CCA_, negative decadic logarithm of allelic *P* values

Subsequently, for a comprehensive fine-mapping of the *FOXO3* gene region, 205 of the detected 1,106 SNVs were analyzed (Supplementary Data 1). These SNVs were either (i) located in regulatory regions (e.g. sites for microRNA binding, DNA methylation or transcription factor binding), (ii) selected through a haplotype-tagging approach or (iii) identified in the exonic regions. The SNVs were investigated in the German case–control sample for association with longevity. Fifty-four nominally statistically significant SNVs (*P* < 0.05) were detected both in the whole German longevity sample (1109 LLI, 95–110 years) and in the smaller subset of 594 centenarians (100–110 years) vs. 918 younger controls (60–75 years) (Supplementary Data 2). As expected, individuals aged 100 years and older yielded larger odds ratios (OR) compared with the younger longevity sample (≥ 95 years; Table 1; Supplementary Data 2). Correction for multiple testing was not deemed necessary since *FOXO3* is already a confirmed longevity gene and since the aim of the association approach was to identify the most promising potentially functional variants. Four of the 54 nominally significant SNVs were located in the non-coding exon 4 (smallest *P* value = 0.003 in the centenarian subsample) (Supplementary Data 2). The in silico analysis with MutationTaster2^20^ did not predict any specific effects for the SNVs in exon 4. Thus, they were not considered for subsequent functional studies. The association signals with the smallest *P* values were observed for the two intronic SNVs rs4946935 (*P*_cent_ = 0.0003, OR = 1.35) and rs12206094 (*P*_cent_ = 0.001, OR = 1.31; Table 1, Fig. 1); therefore, these two SNVs were investigated in more detail. Both are common SNVs, with minor allele frequencies of about 30% in controls and up to 37% in centenarians (Table 1). Based on transcript NM_201559, SNV rs12206094 is located in the second, and rs4946935 in the third (=last) intron of *FOXO3* (Fig. 1). Although the two SNVs are 94.5 kb apart from each other, they are in moderate LD (*r*^2^ = 0.61 based on HapMap-CEU individuals, 1000 Genomes^21,22^). In a model incorporating both SNVs, a significant negative interaction between them was found (Table 2; genotype model: *P* = 0.0011, allelic model: *P* = 0.000018). The ORs for combinations of the favorable longevity (minor) alleles were much reduced due to the interaction effect. It follows that being homozygous for the favorable allele of one SNV and for the unfavorable allele (major) of the other SNV is more advantageous than being heterozygous or even homozygous for the favorable allele of both SNVs. Using the 1000 Genomes Pilot 1 data^21,22^, we estimated the LD between our two variants and rs10457180 which was previously reported to be the most robustly longevity-associated SNV in *FOXO3*^4^. While rs12206094 only showed modest LD with rs10457180 (*r*^2^ = 0.64), the LD between rs10457180 and rs4946935 was stronger (*r*^2^ = 0.92). In Supplementary Data 2, rs10457180 is listed as one of the 54 associated SNVs; however, it had a smaller OR (OR = 1.171 in German LLI vs. younger controls) than our two top-associated SNVs. Therefore, it may well be that the effects of rs4946935 and rs12206094 were tagged by rs10457180.

**Table 1:** Association statistics for the *FOXO3* SNVs rs4946935 and rs12206094 in the whole German study population and centenarian subpopulation

SNV	Study population	MAF^a^ LLI	MAF^a^ Controls	*P*_CCA_^b^	OR^c^	95% C.I.^d^
*Whole study population*						
rs4946935						
	Total	0.349	0.304	0.003	1.228	1.072–1.406
	Female	0.350	0.313	0.037	1.181	1.010–1.381
	Male	0.347	0.278	0.022	1.375	1.046–1.808
rs12206094						
	Total	0.325	0.287	0.010	1.196	1.044–1.369
	Female	0.322	0.291	0.068	1.157	0.989–1.353
	Male	0.333	0.275	0.045	1.318	1.006–1.726
*Centenarian subpopulation*						
rs4946935						
	Total	0.370	0.304	0.0003	1.345	1.147–1.577
	Female	0.372	0.313	0.004	1.303	1.088–1.559
	Male	0.360	0.278	0.032	1.458	1.032–2.060
rs12206094						
	Total	0.345	0.287	0.001	1.306	1.116–1.529
	Female	0.342	0.291	0.011	1.261	1.055–1.508
	Male	0.357	0.275	0.024	1.469	1.052–2.050

Listed are allele frequencies, allelic *P* values, odds ratios, and the 95% confidence intervals for the whole study population and centenarian subpopulation (1109 LLI ≥ 95 years including 594 centenarians, 918 younger controls (age range: 60–75 years)). The statistics are listed for all individuals of the study populations as well as for males and females separately (males: 275 LLI ≥ 95 years including 119 centenarians, 237 younger controls; females: 834 LLI ≥ 95 years including 475 centenarians, 681 younger controls)

^a^ Minor allele frequency, MAF; the definition of the minor allele is based on controls; minor alleles are A for rs4946935 and T for rs12206094

^b^ Allelic *P* values, *P*_CCA_; calculated with chi-squared test with one degree of freedom

^c^ Odds ratio for longevity, OR; based on the minor allele in controls

^d^ 95% confidence interval, 95% C.I.; C.I. for the odds ratio

**Table 2:** Odds ratios of the combined genotypes of rs12206094 and rs4946935

	rs12206094			
		CC	CT	TT
rs4946935	GG	1, *n* = 550	1.81, *n* = 107	3.27, *n* = 2
	AG	1.81, *n* = 69	1.92 (3.28), *n* = 517	2.04 (5.92), *n* = 35
	AA	3.29, *n* = 7	2.05 (5.95), *n* = 28	1.28 (10.76), *n* = 104

Given are the odds ratios (ORs) for the allelic interaction model (multiplicative OR scale). In brackets are the theoretic combined ORs if no interaction were present (given the same single SNV ORs). The underlying population is the German longevity sample with 594 centenarians and 918 younger controls. *n* = number of individuals

### Replication of associations for rs4946935 and rs12206094

The associations for rs4946935 and rs12206094 were confirmed in a French sample (1,264 LLI, 91–115 years; 1,830 younger controls, 35–62 years) with *P = *0.022 (OR = 1.14) and *P = *0.008 (OR = 1.16), respectively (Table 3). In a smaller Danish sample (643 LLI, 92–101 years; 746 younger controls, 56–71 years), the signal at rs12206094 replicated as well (*P = *0.012, OR = 1.24) and the allelic effect of rs4946935, though not statistically significant, showed the same trend as in the French and German individuals (*P = *0.127, OR = 1.14; Table 3). A meta-analysis of all three study populations strengthened the associations (Table 3). The association signals at both SNVs consistently had a higher estimated OR in males than in females (Table 1, Table 3); this difference was significant for rs12206094 in the meta-analysis (*P* = 0.042), but not for rs4946935 (*P* = 0.218). A similar observation was already reported before^7^. The biological implications of these findings remain to be clarified.

**Table 3:** Association statistics for the SNVs rs4946935 and rs12206094 in the French and Danish replication cohorts, respectively

SNV	Study population	MAF^a^ LLI	MAF^a^ Controls	*P*_CCA_^b^	OR^c^	95% C.I.^d^
*rs4946935*						
French	Total	0.324	0.297	0.022	1.136	1.018–1.268
	Female	0.318	0.294	0.094	1.118	0.981–1.274
	Male	0.355	0.302	0.032	1.273	1.020–1.589
Danish	Total	0.304	0.278	0.127	1.137	0.964–1.340
	Female	0.308	0.296	0.568	1.059	0.870–1.289
	Male	0.290	0.248	0.183	1.237	0.905–1.691
Meta-analysis: German, French, Danish	Total	0.329	0.295	2.38E−05	1.188	1.097–1.287
	Female	0.327	0.300	0.003	1.154	1.050–1.267
	Male	0.336	0.285	0.001	1.305	1.110–1.535
*rs12206094*						
French	Total	0.326	0.294	0.008	1.160	1.040–1.295
	Female	0.317	0.291	0.064	1.131	0.993–1.289
	Male	0.364	0.298	0.008	1.345	1.079–1.677
Danish	Total	0.305	0.262	0.012	1.235	1.047–1.458
	Female	0.302	0.282	0.328	1.104	0.905–1.346
	Male	0.315	0.232	0.008	1.527	1.118–2.085
Meta-analysis: German, French, Danish	Total	0.325	0.285	1.31E−06	1.219	1.125–1.321
	Female	0.319	0.289	0.002	1.164	1.059–1.280
	Male	0.348	0.278	1.49E−05	1.426	1.215–1.675

Listed are allele frequencies, allelic *P* values, odds ratios, and the 95% confidence intervals for the whole study populations (1264 LLI ≥ 91 years and 1830 younger controls (35–62 years) from France and 643 LLI ≥ 92 years and 746 younger controls (56–71 years) from Denmark) as well as for the subgroups of females (1032 LLI and 1,105 younger controls from France and 493 LLI and 456 younger controls from Denmark) and males (232 LLI and 725 younger controls from France and 150 LLI and 290 younger controls from Denmark)

^a^ Minor allele frequency, MAF; the definition of the minor allele is based on controls; minor alleles are A for rs4946935 and T for rs12206094

^b^ Allelic *P* values, *P*_CCA_; calculated with chi-squared test with one degree of freedom

^c^ Odds ratio for longevity, OR; based on the minor allele in controls

^d^ 95% confidence interval, 95% C.I.; C.I. for the odds ratio

In addition, we evaluated associations of both SNVs with age-related phenotypes in the Danish study sample (Supplementary Tables 2–5), for which this information was available. Some nominally significant association results were observed (Supplementary Tables 2 and 4). However, after correction for multiple testing, none of them remained significant. When taking into consideration all-cause mortality, none of the identified SNVs showed a significant association. This lack of association with mortality indicates that the two loci contribute to reaching old age, but not to escaping mortality among the extremely long-lived, at least not in the Danish population. This observation is supported by a previous study^7^.

Given the strong and validated associations for rs4946935 and rs12206094 with longevity, we decided to examine the functional influence of these two intronic SNVs.

### SNV allele-specific transcription factor binding affinities

**Fig. 2:**
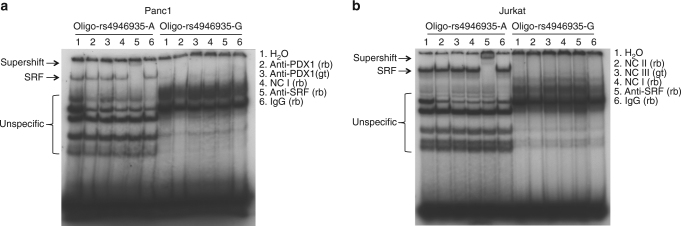
SRF binds to the longevity allele A but not to the major allele G of rs4946935. Nuclear extracts from Panc1 **a** and Jurkat **b** cells were submitted to EMSA with the indicated oligonucleotides. Supershift experiments were performed with antibodies as listed. The position of the supershifted complex is indicated by an arrow. One biological replicate of four is shown. Gt goat, IgG immunoglobulin G, NC nonspecific control (NC I: anti-STAT5A, rabbit; NC II: anti-PDX1, rabbit; NC III, anti-PDX1, goat); PDX1 pancreatic and duodenal homeobox 1, rb rabbit, SRF serum response factor

Bioinformatic analysis suggested allele-specific transcription factor binding for rs4946935. The tool sTRAP^23^ (http://trap.molgen.mpg.de/cgi-bin/trap_two_seq_form.cgi) predicted a high binding affinity of the longevity allele A to serum response factor (SRF, *P = *6.02E − 06, log affinity value 3.37) and of the major allele G to pancreatic and duodenal homeobox 1 (PDX1, *P = *6.02E − 06, log affinity value 4.38). These values for rs4946935 were the most significant ones amongst all 54 nominally associated variants when tested with sTRAP. HaploReg v4.1^24^ and RegulomeDB v1.1^25^ confirmed the sTRAP predictions only for SRF and the A-allele, not for PDX1 and the G-allele. We examined several tissues and cell lines for co-expression of the transcription factors *FOXO3*, *SRF*, and *PDX1* and selected the Panc1 cell line (human pancreatic ductal epithelioid carcinoma cells) as our cellular model, according to the observed expression results (Supplementary Fig. 3). Subsequently, we tested the in silico prediction experimentally by electrophoretic mobility shift assay (EMSA). Appearance of a DNA/protein complex specific for rs4946935-A validated the predicted binding of the transcription factor SRF to the DNA sequence containing rs4946935-A (Fig. 2a). Supershift of this complex by addition of anti-SRF antibody (indicated as “supershift”) specifically confirmed the presence of SRF in the detected DNA/protein complex. Consistent with this result, a binding of SRF to rs4946935-A was also observed in the human T cell leukemia lymphoblast cell line Jurkat (Fig. 2b). As expected from the bioinformatic analysis, no such binding to the major allele G was observed (Fig. 2). Furthermore, neither did the EMSA experiments in Panc1 cells show binding of the transcription factor PDX1 to the DNA sequence containing rs4946935-G or rs4946935-A (Fig. 2a).

For rs12206094, only the tool sTRAP^23^ suggested binding, albeit weak, of the signal transducer and activator of transcription 5 A (STAT5A) to the minor longevity allele (T) (*P = *0.0001, log affinity value −2.03). Since Jurkat cells do not express *STAT5A* (Supplementary Fig. 3), the potential binding was tested in Panc1 cells only. However, supershift experiments with an STAT5A antibody did not show any binding of STAT5A to the DNA sequence containing rs12206094-C or rs12206094-T (Supplementary Fig. 4a, b). It should be noted that we cannot rule out the possibility that other STAT family proteins with similar consensus binding sites bind to this sequence.

According to the Ensembl gene annotation system^26^, rs12206094 is located within a CCCTC-binding factor (CTCF) binding site. Upload of *FOXO3* sequences containing rs12206094-C or rs12206094-T to the CTCF binding site database (CTCFBSDB) 2.0^27^ confirmed a potential binding site for CTCF when the rs12206094-C allele is present. In contrast, the prediction of a CTCF binding site was much weaker for rs12206094-T (Supplementary Table 6). In EMSA experiments using nuclear extracts from Jurkat cells, CTCF binding to the oligonucleotide containing the rs12206094-C allele (Supplementary Fig. 5a, lane 1, right gel) was stronger than to the rs12206094-T-oligonucleotide (Supplementary Fig. 5a, lane 1, left gel). Specificity of CTCF binding was confirmed by supershift experiments (Supplementary Fig. 5a, lane 3). Densitometric quantification of the specific bands against loading control confirmed these observations in four independent experiments (Supplementary Fig. 5b). Despite considerable CTCF expression (Supplementary Fig. 3), no DNA binding of CTCF was observed in Panc1 cells.

### SNV allele-specific influence on promoter activity

**Fig. 3:**
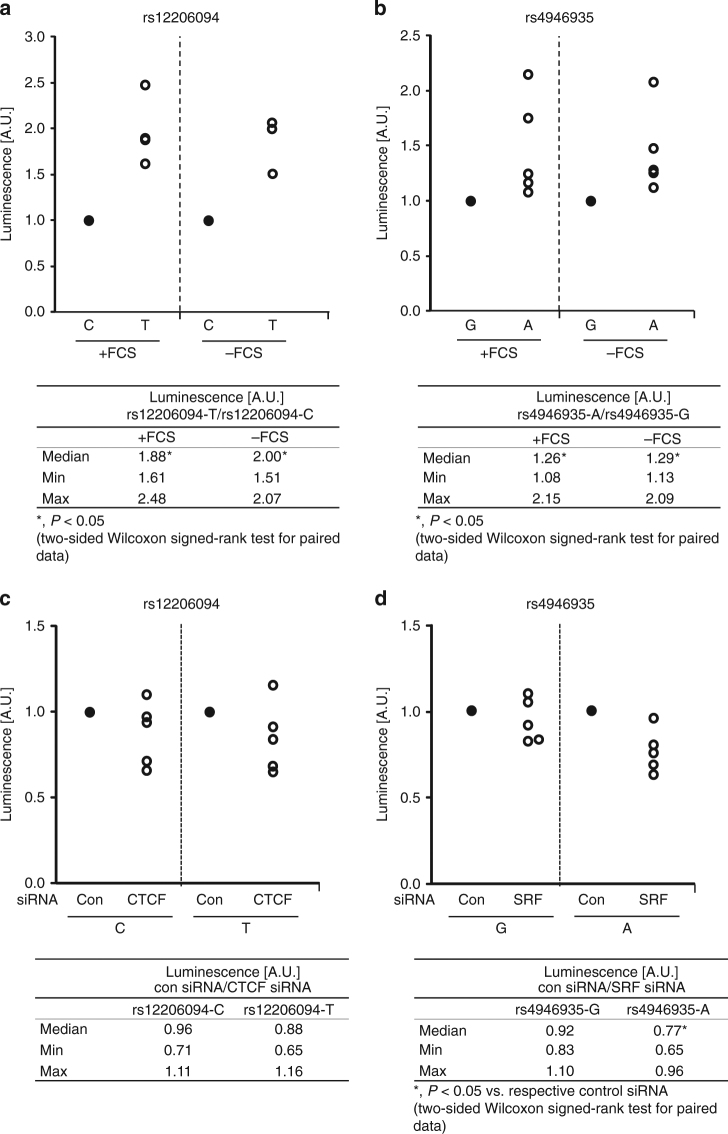
*FOXO3* SNVs rs12206094 and rs4946935 influence luciferase promoter activity, and knockdown of SRF links this transcription factor to rs4946935. **a**, **b** Luciferase promoter assays were perfomed in Panc1 cells transfected with allele-specific constructs for rs12206094 (**a**) and rs4946935 (**b**) in both the presence and absence of FCS. For both SNVs, the promoter activity in cells transfected with constructs containing the respective major allele was set = 1 (black dot). Each white dot represents one independent experiment (*n* = 5). The tables below the figures show the median as well as the minimum and maximum values for the ratio of the longevity allele to the respective alternative allele, taking into account all experiments. **c**, **d** Knockdown of transcription factors was done using specific siRNAs. The luciferase activity in cells treated with control siRNA (con siRNA) instead of the transcription factorspecific siRNA was set = 1 (black dot) for each allele of each SNV. Each white dot represents one independent experiment (*n* = 5). The tables below the figures show the median as well as the minimum and maximum values for the ratios of the activity in presence of con siRNA and transcription factor-specific siRNA for each allele, taking into account all experiments. **a**–**d** For determination of specific luciferase activity, activity of the firefly luciferase was normalized to the activity of the renilla luciferase. *P* ≤ 0.05 was considered statistically significant (two-sided Wilcoxon signed-rank test for paired data). A.U., arbitrary luminescence units

To investigate whether rs4946935 and rs12206094 influence promoter activity in an allele-specific manner, luciferase promoter assays with SNV-specific reporter constructs were performed in Panc1 and Jurkat cells. For both SNVs, luciferase assays showed significantly enhanced promoter activity in Panc1 cells transfected with constructs containing the minor (=longevity) alleles compared with constructs containing the major alleles (*P* < 0.05) (Fig. 3a, b). This effect was seen both in the presence and absence of fetal calf serum (+/−FCS), the latter mimicking nutrient deprivation (Fig. 3a, b). Similar results were observed in Jurkat cells (Supplementary Fig. 6a, b).

To study whether promoter activation by EGCG occurs in an allele-specific manner (as mentioned above, EGCG was shown to modulate *FOXO3* expression^19^), Panc1 and Jurkat cells were transfected with either of the four different *FOXO3* luciferase constructs and additionally treated with EGCG (50 µM). An enhancing effect of this treatment was observed in the absence of FCS for rs12206094-T in both cell lines, reaching statistical significance in Jurkat cells (Supplementary Fig. 7a, c).

To validate that the enhancing effects of the longevity alleles on promoter activity were in fact mediated by allele-specific binding of SRF and CTCF, respectively, we performed luciferase assays with Panc1 cells after siRNA-mediated knockdown of either transcription factor (for verification of the knockdown refer to Supplementary Fig. 8). For rs12206094, no allele-specific effect of the CTCF knockdown on luciferase promoter activity was evident (Fig. 3c). However, in cells transfected with either rs4946935-A or -G, knockdown of SRF reduced the promoter activity when the longevity allele A was present (*P* < 0.05; Fig. 3d).

**Fig. 4:**
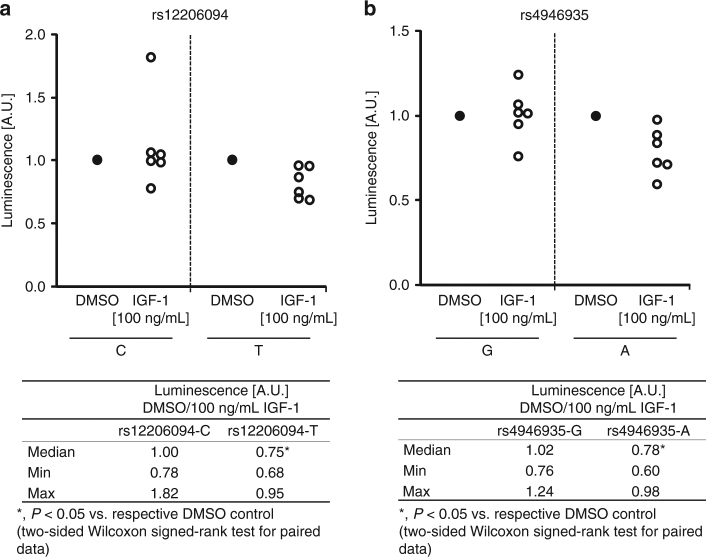
IGF-1 treatment reduces luciferase promoter activity in the presence of the longevity alleles of rs12206094 and rs4946935, respectively. **a**, **b** Luciferase promoter assays were perfomed in Panc1 cells transfected with allele-specific constructs for rs12206094 (**a**) and rs4946935 (**b**). The activity of cells treated with DMSO as control instead of IGF-1 was set = 1 (black dot) for each allele of each SNV. Each white dot represents one independent experiment (*n* = 6). Final DMSO concentrations did not exceed 0.1%. The tables below the figures show the median as well as the minimum and maximum values for the ratios of the activity in presence of DMSO and 100 ng/mL IGF-1 for each allele, taking into account all experiments. For determination of specific luciferase activity, activity of the firefly luciferase was normalized to the activity of the renilla luciferase. *P* ≤ 0.05 was considered statistically significant (two-sided Wilcoxon signed-rank test for paired data). A.U., arbitrary luminescence units

To further investigate the previously reported link between *FOXO3*, IGF-1 levels, and longevity^28^, we treated Panc1 cells (being transfected with either promoter construct and cultured under FCS-deprived conditions) with 100 ng/mL IGF-1. Interestingly, the longevity alleles of both SNVs appeared more susceptible to IGF-1 treatment in terms of a reduced promoter activity than the respective major alleles, which remained rather unresponsive (Fig. 4a, b). Since both SNVs showed the same direction of effect to IGF-1 treatment in our experiments, different mechanisms may mediate this response.

Stimulation with insulin did, in contrast to IGF-1 treatment, not induce consistent allele-specific effects on promoter activity neither at high nor at low concentrations (1 µg/mL and 10 µg/mL, respectively; Supplementary Fig. 9).

### SNV-eQTL associations

To examine whether rs12206094 and rs4946935 or SNVs in LD (*r*^2^ > 0.8 based on HapMap-CEU individuals, 1000 Genomes^21,22^) influenced *FOXO3* gene expression, the publicly available Blood eQTL browser^29^ (http://genenetwork.nl/bloodeqtlbrowser/) and the GTEx eQTL database (http://www.gtexportal.org/home/) were queried. Using the “Test Your Own SNP-Gene Associations” section of the GTEx database, which allows searching for associations between a certain SNV and a gene of interest, we observed statistically significant eQTL associations between *FOXO3* and rs12206094 or rs4946935 in various tissues (several brain regions, pancreas, prostate, testis; Supplementary Table 7). For almost all significant eQTLs, *FOXO3* mRNA expression was higher in the presence of the longevity (minor) alleles for both SNVs (Supplementary Table 7, Supplementary Fig. 10). Further significant cis eQTL associations outside the *FOXO3* gene region were observed for both of the two SNVs or their LD-SNVs in diverse human tissues including blood (Supplementary Table 7).

### SNV allele distributions worldwide

The longevity-associated T-allele of rs12206094 (C/T) represents the derived status and is less frequent than the ancestral C-allele in all investigated populations from the 1000 Genomes Project^22^ (Supplementary Table 8). In contrast, rs4946935 (A/G), with A being both the ancestral and longevity-enabling allele, showed a markedly different distribution. In particular, frequencies between 63.6% and >90% for the A-allele in Subsaharan African populations contrasted with frequencies less than 50% in all non-African populations, namely 26.5–34.1% in Europeans and 17.1–19.2% in Asians (Supplementary Table 8), yielding substantial fixation indices (e.g., *F*_ST_ value 0.48 for CEU vs. LWK and 0.68 for CEU vs. YRI; http://jjwanglab.org/dbpshp)^30^. Such differences may be indicative of either a past selection event, possibly during the initial out-of-Africa migration, or ongoing selection. However, investigation of possible selection in the *FOXO3* gene region yielded inconclusive results. Tajima’s D values (measuring the degree of deviation from neutral evolution), which are based on a single-variant single-population assessment, ranged between 0.96–2.09 and 1.61–3.57 for rs12206094 and rs4946935, respectively, in Europeans from the 1000 Genomes Project^22^ and were substantially higher than those in the East Asian and African populations (Supplementary Table 9). These values in Europeans are consistent with balancing or diversifying selection, but may also mirror a sudden population contraction or one or more admixture events in the past. However, Tajima’s D values for rs12206094 and rs4946935 were by no means exceptional in the light of the general upward shift of Tajima’s D values in all investigated populations (Supplementary Fig. 11, Supplementary Table 9).

## Discussion

In the present study, we report on the functional characterization of the two intronic *FOXO3* SNVs rs4946935 (A/G) and rs12206094 (C/T). The two SNVs yielded the strongest association signals for longevity in the German sample and were therefore chosen for replication in French and Danish longevity collections and functional follow-up studies. Both rs4946935 and rs12206094 were also significantly associated with longevity in the French sample examined here. This is especially re-assuring as in a previous study, three *FOXO3*-longevity associations observed in Germans could not be confirmed in French. However, these discrepancies were most likely due to the much smaller French sample (535 centenarians *vs*. 553 controls) used at that time^6^. In the investigated Danish sample (of likewise modest size), rs12206094 replicated as well, whereas results for rs4946935 were supportive but did not reach statistical significance. Nevertheless, rs4946935 was earlier reported to be associated with longevity in Ashkenazi Jewish centenarians and US American women of European ancestry^8^. In an epidemiological cohort study of white US Americans (aged ≥ 65 years), carriers of the rs4946935-AA genotype had an increased lifespan compared to those with the GA or the GG genotype (1.2 and 1.6 years, respectively)^31^. In the LLI investigated here, the ORs for the two SNVs were, as expected, relatively small (Tables 1 and 3), suggesting a rather modest effect that increased substantially in the subset of centenarians. In addition, both *FOXO3* longevity alleles had a higher estimated OR in males; especially rs12206094-T showed a very strong effect in nonagenarian and centenarian men as observed previously in a Danish sample partly overlapping with the one analyzed here^7^.

The analysis of the two top-associated SNVs with regard to age-related phenotypes in the Danish study population (Supplementary Table 5) yielded no significant associations after correction for multiple testing (Supplementary Tables 2–4). Basic cognitive and physical measures like the cognitive composite score, the Mini-Mental State Examination (MMSE) and chair stand are generally known to show considerable variation in the oldest-old and are predictive of survival^32^. The fact that we did not observe any robust significant associations for these phenotypes with our variants may be a consequence of limited sample size. However, some nominally significant associations were detected that could be worth following up in larger sample collections in future studies.

In model organisms, the suppression of the IIS pathway seems to be the most efficient intervention to extend lifespan^33^. The IIS reduction is coupled to the expression of various members of the FOXO protein family (reviewed in refs ^14,15,33^). It has been hypothesized that a similar mechanism may also operate in humans, likely involving *FOXO3*^13,16^. Our luciferase promoter assays with SNV-specific reporter constructs showed that the minor alleles of rs4946935 and rs12206094, which were more common in centenarians, both considerably enhanced promoter activity in Panc1 and Jurkat cells. Thus, it may be possible that rs4946935-A and rs12206094-T each lead to a reduction in IIS. Surprisingly, when both longevity (minor) alleles were present in the same individual, they genetically interacted in a negative manner, showing much smaller ORs than if they acted independently (Table 2). In future experiments, the interaction effect could be investigated in cells that differ only in the bases at the two SNV positions. These cells might be generated by gene editing with CRISPR/cas9. It is conceivable that due to the combined effect of both longevity alleles on the IIS, the activity of this pathway may be too low to support survival into old age at the best possible rate. Moreover, these two SNVs are probably not the only functionally relevant variations in *FOXO3*. In line with our results, it has already been suggested that a fine-tuned optimal range of IIS is needed for longevity^34^.

Interestingly, also under limited nutrient availability (i.e., absence of FCS in our cell culture models), the longevity alleles of both SNVs caused a higher promoter activity than the major alleles. *FOXO3* is a sensor of nutrient stress^13^ and adverse environmental stimuli (dietary restriction, absence of IGF-1, insulin or growth factors) are known to lead to an increased activity or expression of *FOXO3* and its target genes in various model systems^17,35–37^, ultimately ensuring survival and thus longevity^13^.

In addition, our luciferase promoter studies revealed that, at least in case of rs12206094, treatment with EGCG may further enhance promoter acitivities, substantiating the observation that *FOXO3* can be activated by this tea component^19^. In our experimental set-up, the effect of EGCG on promoter activity was observed under FCS-deprived culture conditions. This could, at least in part, be due to the flavonoid EGCG being bound to serum components in the presence of FCS^38^.

The G to A transition of SNV rs4946935 created a binding site for the transcription factor SRF, which we confirmed experimentally. Our knockdown experiments in Panc1 cells transfected with the rs4946935 allele-specific constructs showed that the enhancing activity of the longevity allele A is mediated by SRF. Interestingly, SRF regulates *Igf-1* expression in various mouse tissues^39,40^, likely through direct binding of SRF to the *Igf-1* promoter^39^. Tissue-specific disruption of the *Srf* gene leads to significant reductions in IGF-1 serum levels^39,40^. MatInspector analysis^41^ revealed SRF binding sites in the human *IGF-1* promoter. Therefore, the binding site in the *FOXO3* intron may compete with that in the *IGF-1* promoter for SRF and thus could also influence *IGF-1* expression. The minor allele of rs2153960, an SNV in strong LD with rs4946935 in Europeans (*r*^2^ = 0.96 based on HapMap-CEU individuals^21,22^), was shown to be associated with both lower serum concentrations of IGF-1 and IGF binding protein 3 (IGFBP-3) as well as with survival beyond 90 years^28,42^. However, to which degree rs4946935-A exerts its effect on lifespan via SRF-binding and eventually lower circulating IGF-1 remains to be clarified.

The CTCF supershift experiments indicated a slightly stronger binding of CTCF to the oligonucleotide containing the major allele C of rs12206094, confirming the CTCFBSDB 2.0^27^ prediction. However, our functional experiments with the rs12206094 allele-specific constructs suggested that the binding strength of CTCF did not influence the luciferase promoter activity. The factor underlying this observation is as yet unclear. Literature data, however, supports a link between *FOXO3* and CTCF binding sites. In 2007, Xie et al.^43^ discovered highly conserved CTCF binding motifs that have regulatory functions. A C → T mutation in one particular motif called LM23, which is very similar to our investigated CTCF binding site, was reported to lead to a considerable reduction in CTCF binding affinity^43^. The C → T transition at rs12206094 may have the same effect. In view of the diverse functions of CTCF, including transcriptional activation, enhancer-blocking and insulator properties and its influence on chromatin organisation^27^, *FOXO3* rs12206094 might affect gene expression of neighboring genes or may even act interchromosomally. Experimental evidence for this hypothesis has recently been published^44^.

In eQTL databases, a higher *FOXO3* mRNA expression was observed in the presence of the rs12206094-T and rs4946935-A longevity alleles for almost all significant eQTLs in various tissues (Supplementary Table 7, Supplementary Fig. 10). Overall, there is support for a decline in *FOXO3* expression during the aging process^13,45^. Furthermore, down-regulation of *FOXO3* was shown to result in cell morphology changes consistent with senescence^46,47^. In line with our results, a higher *FOXO3* mRNA expression was observed before for the longevity-associated G allele of rs2802292 in human skeletal muscle biopsies^16^. These findings, together with the higher promoter activity in our luciferase assays, are in agreement with the observation that a higher *FOXO3* mRNA expression leads to longevity in model organisms^13^. However, in a recent article by Peters et al.^48^ it was shown that *FOXO3* expression increases in human blood with age. It is conceivable that *FOXO3* expression is upregulated during aging to compensate increases in stress caused by old age itself. These heterogeneous observations highlight the complexity of *FOXO3* regulation and may indicate tissue-specific and/or stress level- and stimuli-dependent mechanisms. Further research is needed to clarify how aging affects *FOXO3* expression in detail and which tissues play pivotal roles for longevity.

The longevity allele A of SNV rs4946935 shows a considerable frequency difference between populations that is unlikely to have been produced by genetic drift (Supplementary Table 8). Although rs4946935-A represents the ancestral state, it is today the minor allele in non-Africans. Our results suggest that balancing or diversifying selection may have played a role in the global distribution of the rs4946935 alleles (and perhaps those of rs12206094 as well). The respective selective pressures remain to be identified, although past population admixture or bottleneck events may also have left their footprint on the spatial allele distribution. However, given the functional effects of rs4946935 and rs12206094 on IIS, a dietary influence seems probable. For instance, European hunter-gatherers and early farmers showed a much higher frequency of the rs12206094 longevity allele (53.7% and 47.5%, respectively) than contemporary Europeans^49^ (Supplementary Table 8). The same was observed for a SNV in strong LD with rs4946935 (rs1935949; *r*^2^ = 0.96: 62.0% and 45.3%, respectively)^49^ (Supplementary Table 8), indicating that the dietary shift during the Neolithic may have led to an increase in the alternative alleles C and G. Interestingly, in our promoter assays, the major alleles of both SNVs were rather unresponsive to IGF-1 treatment, while the longevity alleles were more susceptible and resulted in a lower promoter activity. IGF-1 is particularly inducible by ingestion of insulinotropic dairy (e.g., milk)^50^, which likely started in the Neolithic when people began to domesticate animals. Thus, the major alleles may have enabled a sufficient *FOXO3* expression in the presence of an IGF-1 overload that is caused by an animal protein-rich diet. In contrast, the longevity alleles appear to be more beneficial under basal nutrient conditions and famine. Similarly, changes of other metabolic genes in farming communities have been interpreted as successful adaptations to the new Neolithic diets and life-style^49^.

Taken together, our study reveals hitherto undescribed mechanistic and evolutionary aspects of the role of *FOXO3* in human longevity. Most genetic association findings involving variants in non-coding regions have remained elusive so far. Against this background, we here present first experimental evidence for a functional link between common intronic variants in *FOXO3* and the longevity phenotype in humans.

## Methods

### German, French and Danish study populations

The total German study population comprised 515 unrelated nonagenarians and 594 centenarians (95–110 years, mean age 99.3 years). The male:female ratio in the sample was about 1:3. The participants were recruited from different geographic regions of Germany and were all of German ancestry. The 918 unrelated younger controls were 60 to 75 years old (mean age: 67.0 years) and were matched for ancestry, sex, and geographic origin within the country. The recruitment of the German sample was partly organized by the PopGen biobank and has been described in detail elsewhere^51^. All participants gave written informed consent to participate in the study. Approval for the project was obtained from the Ethics Committee of the Medical Faculty of Kiel University.

French centenarians were recruited when they were in their 100th year or beyond^52^. French siblings were recruited when at least two siblings fulfilling the age criterion of 90 years or older were alive in a family. The oldest sibling was selected for the association study. The mean age of centenarians and the group of oldest siblings drawn from each sib pair was 104 and 100 years, respectively (age at death or age at last contact). In total, 1264 unrelated elderly were included in the study (91–115 years, mean age 102.4 years; male:female ratio ~ 1:4.5). All subjects signed a written informed consent form in accordance with the local review board Paris Saint-Antoine. The 1830 French unrelated controls (35–62 years, mean age 49.1 years) were selected in a population-based sample of French subjects that had participated in the Supplementation in Vitamins and Mineral Antioxidants (SU.VI.MAX) study^53^.

The 643 unrelated Danish LLI (92–101 years; mean age 95.7 years; male:female ratio ~ 1:3) were drawn from three population-based nation-wide birth cohort studies conducted at the University of Southern Denmark: The Danish 1905 birth cohort study, the Danish 1910 birth cohort study, and the Danish 1915 birth cohort study^54^. Briefly, the 1905 birth cohort study was initiated in 1998, when participants were 92–93 years of age, and the 1910 and the 1915 Birth Cohort Studies were initiated in 2010, when participants were 100 and 95 years of age, respectively. The 746 unrelated younger controls (56–71 years, mean age 63.1 years; male/female ratio ~ 1:1.5) were drawn from the Study of Middle Aged Danish Twins (MADT)^55^. The sample partly overlapped with the one used in the study by Soerensen et al.^7^. Written informed consent was obtained from all participants and all studies were approved by The Regional Scientific Ethical Committees for Southern Denmark.

### Sequencing and genotyping

For sequencing and genotyping, genomic DNA was extracted from EDTA whole blood (Invisorb Blood Universal Kit; STRATEC Biomedical AG, Birkenfeld, Germany) and subjected to whole genome amplification (following the standard protocol of GenomiPhi V2 Amp Kit 500 RXS; Amersham Biosciences; GE Healthcare Europe GmbH) prior to the genotyping experiments. Three different sequencing approaches were applied: (1) sequencing by ligation (SBL, SOLiD); (2) sequencing by synthesis (SBS, Illumina); and (3) Sanger sequencing. Supplementary Table 1 provides an overview of the samples used in the three sequencing approaches.

To avoid sequencing and genotyping errors that might derive from the *FOXO3* pseudogene *ZNF286B* (99% sequence homology with exonic *FOXO3* regions), we consistently applied a published method to ensure *FOXO3* specifity in the highly homologous exonic regions^10^. For SOLiD sequencing by ligation (SBL), 23 long-range PCR fragments were generated to cover and enrich the whole ~ 170 kb genomic *FOXO3* region. Fragment libraries (50 bp single-end reads) were constructed for 34 German centenarians (≥100 years), 16 younger German controls (64–75 years), and six HapMap-CEU samples (Utah residents with Northern and Western European ancestry; GM12752, GM12753, GM12755, GM11997, GM10838, GM10839). Library preparation and sequencing were performed according to Melum et al.^56^. The obtained reads were mapped using the default parameters of the BioScope Software (Thermo Fisher Scientific Inc., Waltham, USA) against the target region that was created using the FASTA NCBI36/hg18 reference sequence. Due to both poor coverage for parts of the *FOXO3* gene and problems in generating the long-range PCR fragments, some gaps remained in the obtained reads (Supplementary Fig. 1a). Mapping of the obtained sequences against the FASTA NCBI36/hg18 reference revealed that approximately 65% of the reads were mapped for all individuals covering over 80% of the target region at 50×. The sequence coverage was computed using the BedTools package^57^, the Genome Analysis Toolkit^58^ (GATK), and the GATK´s CombineVariants^58^. SNVs were detected using the software programs DiBayes (Thermo Fisher Scientific Inc., Waltham, USA) and SAMtools^59^. The variants were annotated with an in-house pipeline. After annotation, false-positive SNV calls were removed with pibase^60^. The sequences of all primers used for the generation of long-range products for the SBL technology are listed in Supplementary Table 10.

For Illumina sequencing by synthesis (SBS), genomic DNA samples of 48 centenarians (≥ 100 years) und 46 younger controls (61–75 years) were subjected to target enrichment^61^ using RainDance Technologies (RainDance Technologies, Inc., Billerica, MA, USA) to generate 349 fragments (300–600 bp; Supplementary Data 3) that covered the whole *FOXO3* region except for exons 3 and 4. For these large exons, two long-range PCR amplicons (2704 bp and 5,523 bp, respectively; Supplementary Table 11) were produced and sequenced to avoid contamination with highly homologous ZNF286B reads^10^. Paired-end sequencing with 2 × 100 bp reads was performed on the Illumina HiSeq 2000 (Illumina, San Diego, CA, USA) according to the manufacturer’s instructions. The sequences were aligned to hg19 using Burrows–Wheeler Aligner with default parameters^62^. Sequence coverage was analysed with the BedTools package^57^ and Picard (picard.sourceforge.net/). Base calling and quality check after sequencing was done with the GA pipeline version 1.3 (support.illumina.com/downloads/pipeline_132.ilmn) and the FastQC package (http://www.bioinformatics.babraham.ac.uk/projects/fastqc/), respectively. Mapping and variant-calling was performed as previously published^63^. Briefly, to prevent false-positive SNV calls at the end of sequencing reads and obtain accurate scores on SNV calls, local realignment around indels and quality score recalibration was done using GATK^58^, non-uniquely mapped reads were removed with SAMtools^59^, duplicates were removed using Picard’s MarkDuplicates (picard.sourceforge.net/), variant calling was performed with SAMtools^59^ and GATK^58^, and the results were subsequently merged for further analysis using GATK’s CombineVariants^58^. False-positive SNV calls were removed with pibase^60^ and annotation was performed with an in-house pipeline.

In another set of 138 LLI (mean age: 101.5 years) and 92 controls (60–75 years), Sanger sequencing (Thermo Fisher Scientific, Waltham, Massachusetts, US) was performed for all four exons, the exon-intron boundaries, and the promoter of *FOXO3* transcripts NM_201559 and NM_001455 to provide additional information on these important regions. Prior to sequencing, 24 PCR amplicons were generated for the investigated *FOXO3* region. For the two large exons (exon 3 and exon 4), *FOXO3-*specific long-range products were used as templates. SNVs were detected and visualized with the software packages novoSNV^64^ and InSNP^65^. The sequences of all primers used for *FOXO3* Sanger sequencing and the generation of long-range products for exon 3 and exon 4 are listed in Supplementary Table 11.

### SNV selection

For extensive fine mapping, altogether 205 SNVs located in the *FOXO3* gene region were genotyped and analyzed in a German case–control association study for longevity. The typed SNVs were mainly selected through a haplotype-tagging approach (based on the HapMap genotypes of Europeans applying Haploview 4.2 (http://www.broad.mit.edu/mpg/haploview/)) to ensure that most of the allelic variation of the genomic regions was captured and the common haplotypes were represented. In addition, SNVs that were located in regulatory regions (microRNA binding sites, methylation sites, enhancer sites, transcription factor-binding sites, degree of conservation, and Dnase I hypersensitive sites) were selected. Furthermore, all detected common and rare SNVs in coding and non-coding exonic regions (*n* = 28) were selected for genotyping due to the functional relevance of these regions. The bioinformatic tools and databases used for SNV selection are listed in the footnote of Supplementary Data 2. For further information on the variants, we applied in silico analysis to the SNVs in the coding exons 2 and 3 with various bioinformatic tools (Supplementary Table 12). For the non-coding exonic SNVs in exon 4, MutationTaster2^25^ was used (Supplementary Table 13). Altogether, no functional relevance was detected for any of the exonic SNVs that were associated with longevity in our data.

For the four SNVs in exon 2, no *FOXO3*-specific long-range products could be generated. Two of the four detected SNVs (rs150320900 and snv_chr6_108989376) showed a very low MAF (MAF ≤ 0.001) and were excluded from the case–control study as the low MAF precluded any meaningful statistical power for association studies. SNV rs11757217 was not considered either for further analysis as the generated *FOXO3*-specific sequencing data showed a frequency difference of less than 2% between 170 centenarians and 136 younger controls. For rs111556510, suitable SNVs in strong LD (*r*^2^ > 0.8) located in the intronic region (rs77626104, rs111254195, rs113032762) were selected for genotyping instead.

### Genotyping

Genotyping of the German, French and Danish samples was performed either by TaqMan (Thermo Fisher Inc., Waltham, Massachusetts, USA) or iPLEX (Sequenom; San Diego, CA, USA) technology. TaqMan genotyping was carried out on a 7900HT Fast Real-time PCR System (Thermo Fisher Scientific Inc., Waltham, MA, USA). The sequenom genotyping was done on a MassARRAY Analyzer 4 System (Sequenom, Hamburg, Germany). Sequenom assays were designed using the Human Genotyping Tool (https://www.mysequenom.com/Tools) and the Typer Analyzer 4.0 Software (Sequenom, Hamburg, Germany). *FOXO3*-specific long-range PCR products served as PCR-templates for SNVs located in exon 3 and 4. Part of the Danish sample was genotyped on the Illumina HumanOmniExpress BeadChips (Illumina Inc., San Diego, CA, USA) followed by imputation using the 1000 G Phase I Integrated Release Version 3 reference panel and the IMPUTE2 program^66^.

### Analysis of transcription factor-binding sites

For the detection of allele-specific transcription factor-binding sites, intronic *FOXO3* regions containing the allelic variants of rs4946935 and rs12206094 were analyzed using HaploReg v4.1^24^ (www.broadinstitute.org/mammals/haploreg/), RegulomeDB^25^ (http://regulome.stanford.edu/), sTRAP^23^ (http://trap.molgen.mpg.de/cgi-bin/trap_two_seq_form.cgi), and the CTCF binding site database (CTCFBSDB) 2.0 (http://insulatordb.uthsc.edu/)^27^.

### Construction of plasmids

The reporter plasmids pGL3_rs4946935-G for the major allele and pGL3_rs4946935-A for the minor allele were generated by the insertion of a 134-bp region into the pGL3 promoter vector (Promega, Madison, WI, USA) upstream of the promoter-luciferase transcriptional unit using specific primers in accordance with the NEB Q5 site-directed mutagenesis protocol (New England BioLabs, Ipswich, MA). pGL3_rs12206094-T (minor allele) was constructed by inserting an 80 bp region using the same strategy. For pGL3_rs12206094-C (major allele) primers with a substitution of the minor allele (TTG C**T**A CCA) to the major allele (TTG C**C**A CCA) were designed. The major allele was inserted by using the QuickChange Side-directed Mutagenesis Kit (Stratagene, La Jolla, USA) according to the manufacturer´s instructions. All constructs were verified by DNA sequencing. The plasmid DNA was purified using the Plasmid Midi Kit (Invitrogen, Carlsbad, CA, USA). The sequences of all primers used in the generation of the plasmids are listed in Supplementary Table 14.

### Electrophoretic mobility shift assay and supershift assay

The human cell lines Panc1 (ATCC via LGC Standards, Wesel, Germany) and Jurkat (kindly provided by Prof. Hendrik Ungefroren, Department of Internal Medicine I, University Hospital Schleswig-Holstein, Campus Lübeck) were maintained at 37 °C, 85% humidity and 5% CO_2_ in RPMI1640 medium supplemented with 10% FCS, 1% L-glutamine. The cell culture media for Panc1 cells was additionally supplemented with 1% sodium pyruvate. Cells were tested regularly by MycoAlert Mycoplasma Detection Kit (Lonza, Cologne, Germany). Preparation of nuclear extracts was done as described previously^67^. For gel-shift assays, 5 µg of nuclear extracts were incubated with ^32^P-labeled oligonucleotides (Supplementary Table 14) spanning the allele-specific putative transcription factor-binding sites (or a known consensus sequence (STAT5A, sc-2565; Santa Cruz, Heidelberg, Germany)) of the indicated transcription factors. For supershift experiments, 4 µg of antibody (anti-SRF (sc335x), anti-PDX1 (sc14664x, goat), anti-STAT5A (sc1081x), anti-CTCF (sc271474x), all Santa Cruz, Heidelberg, Germany; anti-PDX1 (5679x, rabbit), anti-IgG (2729, rabbit), both New England Biolabs GmbH, Frankfurt a.M., Germany); anti-IgG (Merck Chemicals GmbH, Darmstadt, Germany)) were additionally added to the reaction mix 30 min prior to electrophoresis and incubated at 4 °C. After separation by gel electrophoresis (100 V, 4 °C), gels were dried and exposed to X-ray Hyperfilm (Amersham, Freiburg, Germany). Densitometry of the CTCF-specific band against immunoglobulin G (IgG) was performed using the ImageJ software (https://imagej.nih.gov/ij/).

### Luciferase assay

To analyze the impact of the different alleles on transcriptional activity, 3 × 10^4^ Panc1 or Jurkat cells were seeded into each well of a 48-well plate (Greiner, Frickenhausen, Germany). For this, cells were resuspended in 500 µL RPMI-complete medium (RPMI-1640 supplemented with 10 % fetal calf serum (FCS), 1 % L-glutamine and 1 % sodium pyruvate, all purchased from Biochrom (Biochrom GmbH, Berlin, Germany)). After culturing overnight, the medium was removed and the cells were transfected with 80 ng per well of either of the firefly constructs together with 40 ng per well of pRL-TK (reporter vector for normalization using *Renilla* luciferase controlled by the herpes simplex virus thymidine kinase (HSV-TK) promoter; Promega, Mannheim, Germany) using the Effectene Transfection Reagent according to the instruction of the manufacturer (Qiagen, Hilden, Germany). The following firefly plasmids were used: pGL3-rs4946935-A (containing the minor allele (A)), pGL3-rs4946935-G (containing the major allele (G)), pGL3-rs12206094-T (containing the minor allele (T)) or pGL3-rs12206094-C (containing the major allele (C)). After an incubation period overnight, the cell culture medium was changed with cells being either cultured in RPMI-1640 complete medium (+FCS) or in RPMI-1640 medium lacking FCS (-FCS). Additionally, cells were treated either with 1 µl EGCG (Sigma-Aldrich Chemie Gmbh, Munich, Germany) at a concentration of 50 µM and an equal volume of DMSO as control, respectively, with 100 ng/mL IGF-1 (Merck, Darmstadt, Germany) or with 1 or 10 µg/mL insulin (Sigma-Aldrich Chemie Gmbh, Munich, Germany). After 48 h, cells were washed once with PBS and lysed for measurement of luciferase activity using the Dual-Luciferase Reporter Assay System according to the manufacturer’s instructions (Promega, Mannheim, Germany). All measurements were performed with a Tecan Infinite M200 PRO luminometer (Tecan, Crailsheim, Germany). For determination of specific luciferase activity, activity of the firefly luciferase was normalized to the activity of the renilla luciferase.

### siRNA-mediated knockdown

To suppress expression of CTCF and SRF in Panc1 cells for luciferase assays, 1 × 10^5^ Panc1 cells per well were seeded in 12-well-plates containing 1 mL medium per well. After 24 h, medium was removed and 1 mL fresh medium was added. Then, 6 µL per well HiperFect reagent (Qiagen, Hilden, Germany) and 2.5 µL of either negative control siRNA (sc-37007, Santa Cruz) or specific siRNA against SRF (sc-36563, Santa Cruz) or CTCF (sc-35124; Santa Cruz), all in a concentration of 25 nM, were mixed with 100 µL FCS-free medium and added to cells. After 72 h, cells were detached, seeded and transfected and treated for luciferase assay as described above.

### RNA isolation and RT-qPCR

Knockdown of CTCF and SRF was verified by RT-qPCR. For this purpose, total RNA of siRNA transfected Panc1 cells was isolated with the total RNA kit peqGOLD (PeqLab, Erlangen, Germany) and subjected to reverse transcription according to the manufacturer’s instructions (Fermentas, via Thermo Fisher Scientific, Darmstadt, Germany). CTCF and SRF primers were purchased from Microsynth AG (Lindau, Germany) and GAPDH primers from Eurofins (Ebersberg, Germany). Primer sequences are: *CTCF* FW: 5′-TCTGACAGTGAAAATGCTGA-3′; RV: 5′-TCTGGTCTTCAACCTGAATG-3′; *GAPDH* FW: 5′-TCCATGACAACTTTGGTATCGTGG-3′; RV: 5′-GACGCCTGCTT-CACCACCTTCT-3′; *SRF* FW: 5′-CTCAACTCGCCAGACTCTCC-3′; RV: 5′-CCGGCTT-CAGTGTGTCCTTG-3′. Primer specificity was tested by melting curve analysis and verification of PCR product size. All PCRs were performed as duplicates with a LightCycler 480 (Roche, Mannheim, Germany) for 50 cycles followed by a melting curve analysis. The amount of RNA of the gene of interest was normalized to the RNA amount of the control gene GAPDH.

### Analysis of tissue-specific expression patterns

Tissue- and cell type-specific expression patterns of *FOXO3*, *SRF*, *STAT5A*, *CTCF*, and *PDX1* were analysed by endpoint-PCR. Analyzed cDNAs of human tissues (brain, heart, lung, skeletal muscle, spleen, thymus, liver, kidney, pancreas, duodenum, jejunum, colon, bone marrow, ovaries and testis) were purchased from Takara Bio Europe SAS (Saint-Germain-en-Laye, France). cDNAs of the investigated cell lines (Panc1; Jurkat; human colon adenocarcinoma cell line, HT-29; human embryonic kidney cell line, HEK; human epithelial carcinoma cell line, Hela) were prepared using standard methods. *GAPDH* was used as endogenous control. Primer sequences for the specific amplification of *FOXO3*, *SRF*, *STAT5A*, *CTCF*, and *PDX1* are listed in Supplementary Table 14. Amplified PCR products were separated on a 1% agarose gel and visualized using the Universal Hood II (Biorad, München, Germany).

### eQTL analysis

For the two best-associated *FOXO3* SNVs rs12206094 and rs4946935 as well as for SNVs in LD with *r*^2^ > 0.8 (based on HapMap-CEU individuals, 1000 Genomes^21,22^), significant cis eQTL associations in whole blood and various tissues were looked up in the publicly available databases GTEx (http://www.gtexportal.org/home/) and Blood eQTL browser^29^ (http://genenetwork.nl/bloodeqtlbrowser/). Using the “Test Your Own SNP-Gene Associations” section of the GTEx database, we computed associations between *FOXO3* mRNA expression and rs12206094 or rs4946935 in all tissues available without Q-value filtering.

### In silico promoter analysis

In silico promoter analysis of the human *IGF-1* promoter was performed using the MatInspector analysis tool of the Genomatix software^41^ (https://www.genomatix.de/cgi-bin/matinspector_prof/).

### Statistical analyses

Univariate statistical analysis of the case–control studies was performed using PLINK v.1.07 (http://pngu.mgh.harvard.edu/~purcell/plink/) software. Allelic *P* values were calculated using the Chi square test with *P* values ≤ 0.05 being considered nominally significant. Odds ratios are always based on minor alleles in controls. SNVs were tested for Hardy–Weinberg equilibrium in the control group with a significance level of 0.001. The LD information was based on HapMap and 1000 Genomes data using the SNAP Proxy Search function^21,22^ (https://archive.broadinstitute.org/mpg/snap/ldsearch.php).

Additionally, we performed logistic regression analysis with longevity as outcome and SNVs as influence variables with the statistical software R, version 3.2.2 (http://www.R-project.org/)^68^. Genotypic (saturated), allelic, dominant, or recessive effects were used for the coding of the SNVs. Since no clear superiority of dominant or recessive modelling could be detected, the genotypic and allelic coding was applied. The SNVs were tested as single influence variables as well as jointly (with and without interaction). Additionally, we tested for an interaction of sex and SNV effect. Because of the logistic regression approach, a significant interaction between two variables means that the joint OR of the two variables is unequal to the multiplication of the single ORs of the variables. Because of a larger and more reliable sample size for the interaction in the allelic model vs. the genotypic one, the resulting allelic ORs are displayed in Table 2.

For a meta-analysis of rs4946935 and rs12206094, we combined the data from the German, French and Danish studies. For the German study, the centenarians and not the LLI were used as cases. A logistic regression was performed and adjusted for population. No significant interaction between population and SNV effect was found. Additionally, we tested for an interaction between sex and variant, adjusting for population and sex.

All statistical analyses for rs4946935, rs12206094, and age-related phenotypes (Supplementary Tables 2–5) in Danish LLI and controls were performed using the statistical software Stata (Stata version 14.2; Stata Corporation, College Station, TX, USA). Linear regression was used for the analysis of age at first child, age at last child, age at menopause, timed chair stand, cognitive composite score, grip strength, and number of biological children. Ordinal logistic regression (proportional odds model) was used for the analysis of chair stand and Mini-Mental-State-Examination. All analyses were adjusted for age at assessment and sex.

Population genetic inferences were based on the Pilot 1 data from the 1000 Genomes Project^22^, available from the International Genome Sample Resource (IGSR; http://www.internationalgenome.org/). Tajima’s D values and *F*_ST_ estimates following Weir & Cockerham^69^ were obtained from the dbPSHP database resource^30^ (http://jjwanglab.org/dbpshp).

For the statistical analysis of the luciferase experiments, the two-sided Wilcoxon signed-rank test for paired data was applied. A significance level of 0.05 was chosen. Calculations were performed in SPSS, version 22. For graphical display, values were normalized.

In total, 205 SNVs from the sequencing experiments were selected for association testing in the German longevity sample of 1109 cases and 918 controls. For an OR of 1.3 and a two-sided test of difference of proportions between cases and controls, this results in a power between 46% (MAF in cases = 0.10) and 84% (MAF in cases = 0.50) for a significance level of 0.05. Since *FOXO3* is already an established longevity gene and the aim of our study was to identify functional variants and not to validate the association, adjustment for multiple testing was not performed.

The variants rs12206094 and rs4946935 were later tested for association also in the French and Danish longevity sample (French: *n*_cases_ = 1264, *n*_controls_ = 1830, Danish: *n*_cases_ = 643, *n*_controls_ = 746). For rs12206094 with a minor allele frequency of 0.325 in cases and an OR of 1.3, this results in a power of 93% in the French and of 64% in the Danish sample (significance level of 0.05). For rs4946935 with a minor allele frequency of 0.349 in cases and an OR of 1.3, the corresponding power is 94% in the French and 66% in the Danish sample (significance level of 0.05). Power calculations were performed with G*Power, version 3.1.9.2^70^.

### Data availability

All samples and information on their corresponding phenotypes were obtained from the PopGen Biobank (Schleswig-Holstein, Germany) and can be accessed through a Material Data Access Form. Information about the Material Data Access Form and how to apply can be found at http://www.uksh.de/p2n/Information + for + Researchers.html.

## Electronic supplementary material

Supplementary Information

Description of Additional Supplementary Files

Supplementary Data 1

Supplementary Data 2

Supplementary Data 3
