# Publisher Correction: Identification and characterization of two functional variants in the human longevity gene FOXO3

**DOI:** 10.1038/s41467-018-02842-8

**Published:** 2018-01-17

**Authors:** Friederike Flachsbart, Janina Dose, Liljana Gentschew, Claudia Geismann, Amke Caliebe, Carolin Knecht, Marianne Nygaard, Nandini Badarinarayan, Abdou ElSharawy, Sandra May, Anne Luzius, Guillermo G. Torres, Marlene Jentzsch, Michael Forster, Robert Häsler, Kathrin Pallauf, Wolfgang Lieb, Céline Derbois, Pilar Galan, Dmitriy Drichel, Alexander Arlt, Andreas Till, Ben Krause-Kyora, Gerald Rimbach, Hélène Blanché, Jean-François Deleuze, Lene Christiansen, Kaare Christensen, Michael Nothnagel, Philip Rosenstiel, Stefan Schreiber, Andre Franke, Susanne Sebens, Almut Nebel

**Affiliations:** 1 Institute of Clinical Molecular Biology, Kiel University, University Hospital Schleswig-Holstein, Campus Kiel, Rosalind-Franklin-Straße 12, 24105 Kiel, Germany; 2 Department of Internal Medicine I, University Hospital Schleswig-Holstein, Campus Kiel, Arnold-Heller-Straße 3, 24105 Kiel, Germany; 3 Institute of Medical Informatics and Statistics, Kiel University, University Hospital Schleswig-Holstein, Campus Kiel, Brunswiker Straße 10, 24105 Kiel, Germany; 4 The Danish Aging Research Center, and the Danish Twin Registry, Epidemiology, Biostatistics and Biodemography, Department of Public Health, University of Southern Denmark, J. B. Winslows Vej 9B, 5000 Odense C, Denmark; 5 Faculty of Sciences, Division of Biochemistry, Chemistry Department, Damietta University, 34511 New Damietta City, Egypt; 6 Institute of Human Nutrition and Food Science, Kiel University, Hermann-Rodewald-Straße 6, 24118 Kiel, Germany; 7 Institute of Epidemiology, Kiel University, University Hospital Schleswig-Holstein, Campus Kiel, Niemannsweg 11, 24105 Kiel, Germany; 8 Centre National de Recherche en Génomique Humaine CNRGH-CEA, 91000 Evry, France; 9 Université Sorbonne Paris Cité-UREN, Unité de Recherche en Epidémiologie Nutritionnelle, U557 Inserm, U1125 Inra, Cnam, Université Paris 13, CRNH IdF, 93000 Bobigny, France; 10 Department of Statistical Genetics and Bioinformatics, Cologne Center for Genomics, University of Cologne, Weyertal 115b, 50931 Cologne, Germany; 11 Institute of Reconstructive Neurobiology and Life & Brain GmbH, University of Bonn, Sigmund-Freud-Straße 25, 53127 Bonn, Germany; 12 Max Planck Institute for the Science of Human History, Kahlaische Straße 10, 07745 Jena, Germany; 13 Fondation Jean Dausset-Centre d’Etude du Polymorphisme Humain (CEPH), 27 Rue Juliette Dodu, 75010 Paris, France; 14 Department of Clinical Genetics, and Department of Clinical Biochemistry and Pharmacology, Odense University Hospital, Sdr. Boulevard 29, 5000 Odense C, Denmark; 15 Institute for Experimental Cancer Research, Kiel University, University Hospital Schleswig-Holstein, Campus Kiel, Arnold-Heller-Straße 3, 24105 Kiel, Germany

Correction to: *Nature Communications* 10.1038/s41467-017-02183-y, published online 12 December 2017

The original version of this Article contained an error in the spelling of the author Robert Häsler, which was incorrectly given as Robert Häesler. This has now been corrected in both the PDF and HTML versions of the Article.

