# Supplementary material for: Identification and characterization of two functional variants in the human longevity gene FOXO3
Source: Nat Commun. 2017 Dec 12;8:2063. doi: 10.1038/s41467-017-02183-y (PMC5727304; doi:10.1038/s41467-017-02183-y)
Supplement: Supplementary file 1 — Supplementary Information [file 41467_2017_2183_MOESM1_ESM.pdf]

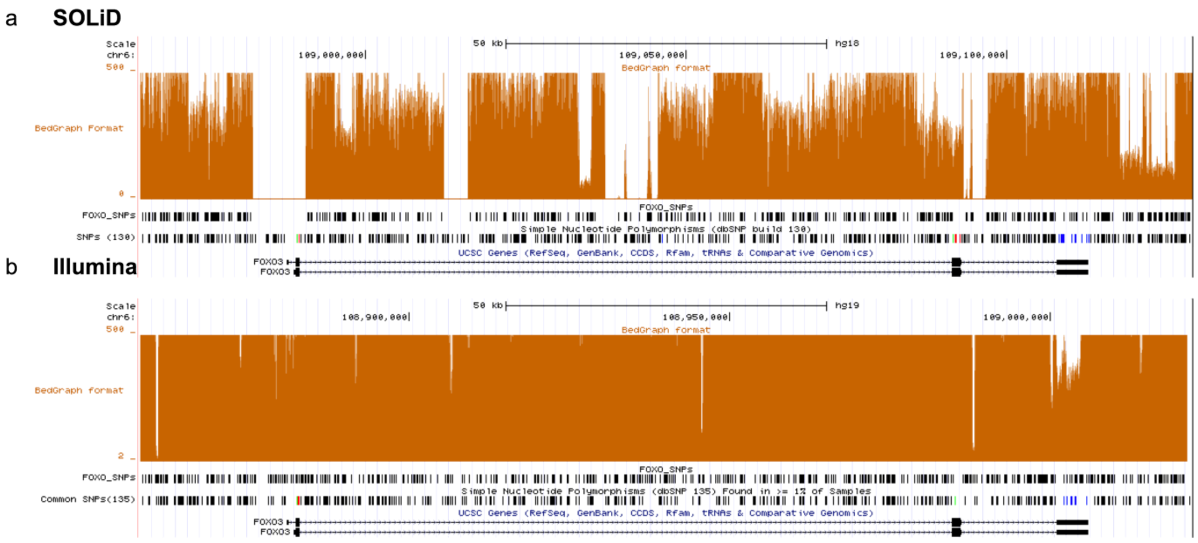

Supplementary Figure 1. Sequence coverage of the *FOXO3* gene region using SOLiD sequencing by ligation (SBL) **(a)** and Illumina sequencing by synthesis (SBS) **(b)** technology. SBL resulted in incomplete sequencing reads in some gene regions due to gaps in the long-range PCR products **(a)**. The regions which remained uncovered applying SBL (mainly before and after the 5´UTR and within the last two exons) were closed with SBS technology **(b)**, which resulted in a fully covered *FOXO3* gene region without any gaps. While with SBL 80% of the target sequence was covered with 50-fold coverage, SBS sequencing yielded a 30-fold coverage of 99% of the target region sequenced. This figure was created using UCSC Genome Browser (http://genome.ucsc.edu/[1]).

FOXO3 resequencing	Sequencing technology		
	**SOLiD** 34 LLI 22 C	**Illumina** 48 LLI 46 C	**Sanger** 138 LLI 92 C
Whole gene region	x	x	-
Promoter	x	x	x
Exons	x	x	x
Number of detected SNVs	817	557	42

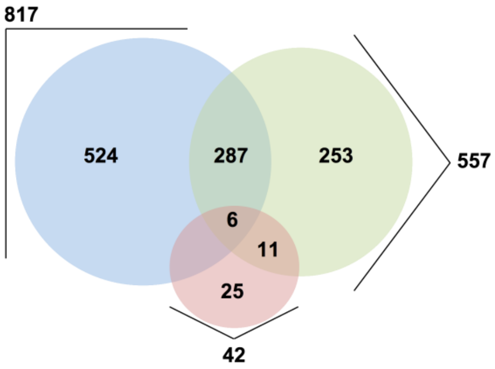

Supplementary Figure 2. Number of detected SNVs in the *FOXO3* gene region using SOLiD sequencing by ligation (SBL, blue), Illumina sequencing by synthesis (SBS, green) and Sanger (red) technology and overlap between the three sequencing technologies. SBL and SBS yielded 293 overlapping SNVs; 524 SNVs were only detected with SBL and 264 SNVs only with SBS. Sanger sequencing yielded 42 SNVs in total; of the 42 SNVs, 17 were also detected with SBS and of these, 6 with all three sequencing technologies. C, younger control group; LLI, long-lived individuals; SNV, single nucleotide variant.

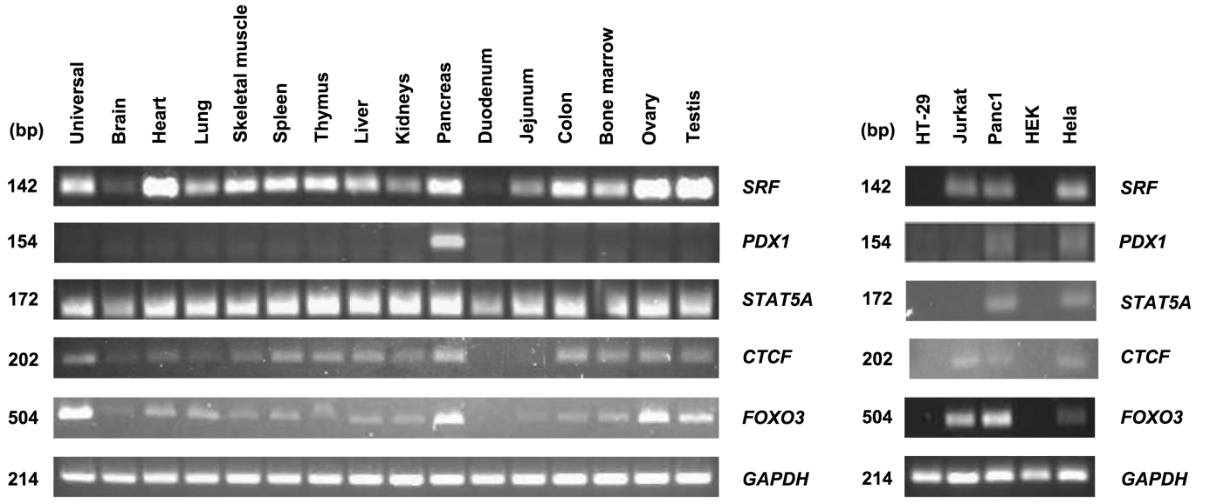

Supplementary Figure 3. Gene expression pattern of *SRF*, *PDX1*, *STAT5A*, *CTCF*, and *FOXO3* in various tissues and cell lines. The expression of *GAPDH* served as control. bp, base pairs; *CTCF*, CCCTC-binding factor; *FOXO3*, forkhead box O3; *GAPDH*, glyceraldehyde-3-phosphate dehydrogenase; HEK, human embryonic kidney cell line; Hela, human epithelial carcinoma cell line; HT-29, human colon adenocarcinoma cell line; Jurkat, human acute lymphocytic leukemia cell line; Panc1, human pancreatic epithelial carcinoma cell line; *PDX1*, pancreatic and duodenal homeobox 1; SRF, serum response factor; *STAT5A*, signal transducer and activator of transcription 5A; universal, mix of 20 different human tissues.

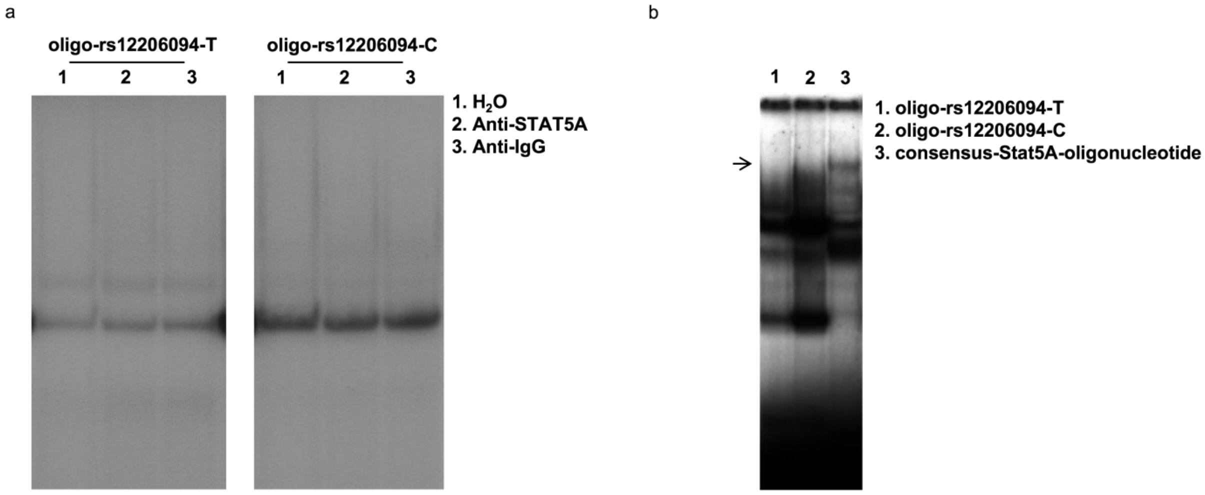

Supplementary Figure 4. STAT5A does not bind to the *FOXO3* rs12206094 alleles. **(a)** Nuclear extracts from Panc1 cells were submitted to EMSA with 32-P labeled oligonucleotides containing the longevity allele T (left) or the major allele C (right) of rs12206094. Supershift experiments were performed with the indicated antibodies. One biological replicate of five is shown. **(b)** A consensus-*Stat5A*-oligonucleotide generates, in contrast to rs12206094 oligonucleotides, a specific band in the EMSA (indicated by an arrow). One biological replicate of three is shown. IgG, immunoglobulin G; STAT5A, signal transducer and activator of transcription 5A.

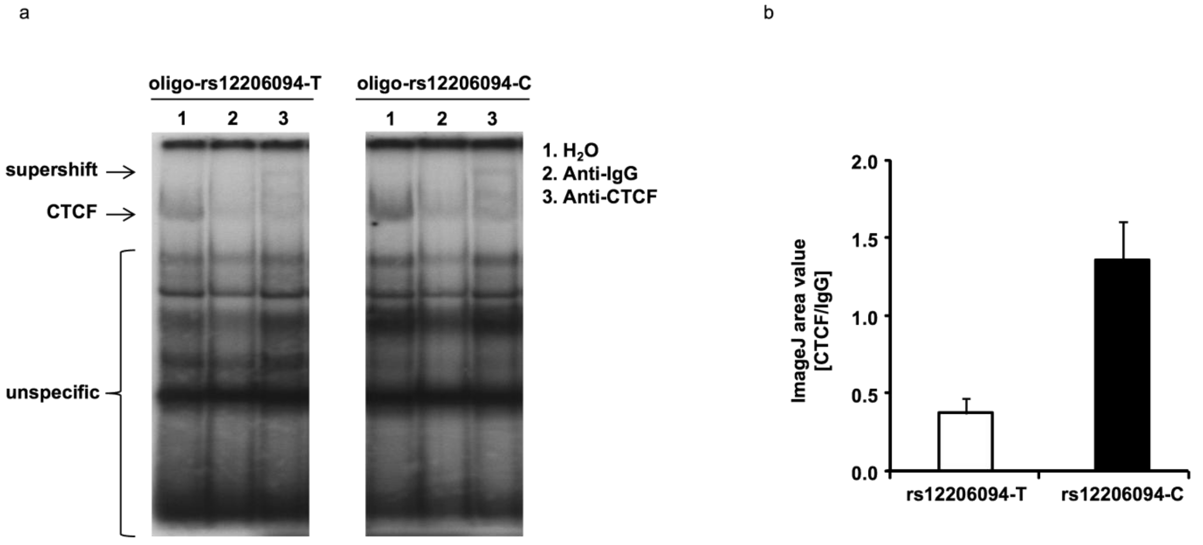

Supplementary Figure 5. CTCF shows stronger binding to the rs12206094-C- than to the rs12206094-T-oligonucleotide. **(a)** Nuclear extracts from Jurkat cells were submitted to EMSA with the indicated oligonucleotides. Supershift experiments were performed with the labeled antibodies. The position of the supershifted complex is indicated by an arrow. One biological replicate of four is shown. **(b)** Densitometry of the CTCF-specific band against control + S.D., n=4 independent experiments. CTCF, CCCTC-binding factor; IgG, immunoglobulin G; S.D., standard deviation.

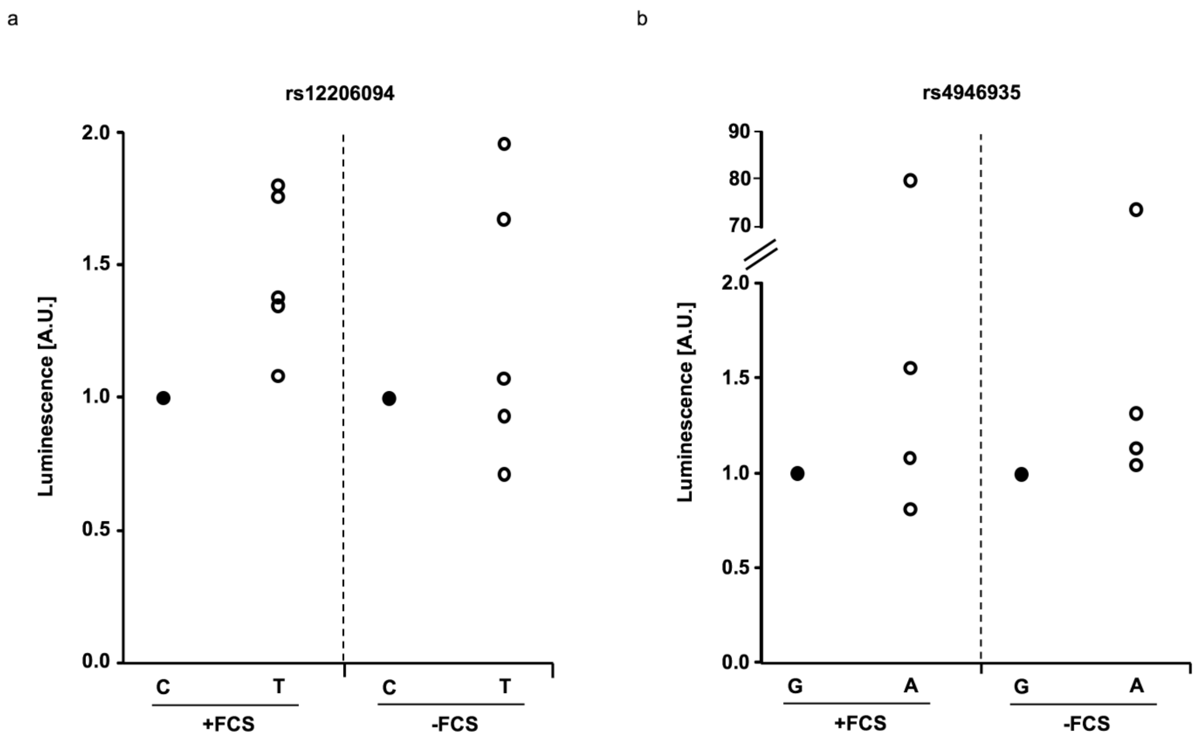

	Luminescence [A.U.] rs12206094-T/rs12206094-C	
	+FCS	-FCS
Median	1.39*	1.08
Min	1.08	0.71
Max	1.81	1.97

*, $P < 0.05$
(two-sided Wilcoxon signed-rank test for paired data)

	Luminescence [A.U.] rs4946935-A/rs4946935-G	
	+FCS	-FCS
Median	1.33	$1.23^{n.s.}$
Min	0.81	1.05
Max	79.8	73.5

n.s., $P = 0.068$
(two-sided Wilcoxon signed-rank test for paired data)

Supplementary Figure 6. In Jurkat cells, the *FOXO3* SNVs rs12206094 and rs4946935 influence luciferase promoter activity in an allele-specific manner either in presence (rs12206094, **(a)**) or in absence (rs4946935, **(b)**) of FCS. **(a, b)** For both SNVs, the promoter activity in cells transfected with constructs containing the respective major allele was set = 1 (black dot). Each white dot represents one independent experiment (6a: n = 5, 6b: n = 4). The tables below the figures show the median as well as the minimum and maximum values of the ratio of the longevity allele to the respective alternative allele, taking into account all experiments. For determination of specific luciferase activity, activity of the firefly luciferase was normalized to the activity of the renilla luciferase. A.U., arbitrary luminescence units.

7a

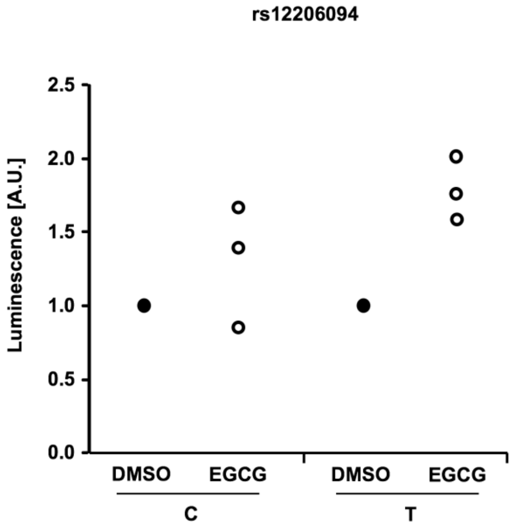

	Luminescence [A.U.] DMSO/50 µM EGCG	
	**rs12206094-C**	**rs12206094-T**
**Median**	1.40	1.77
**Min**	0.86	1.59
**Max**	1.68	2.03

7b

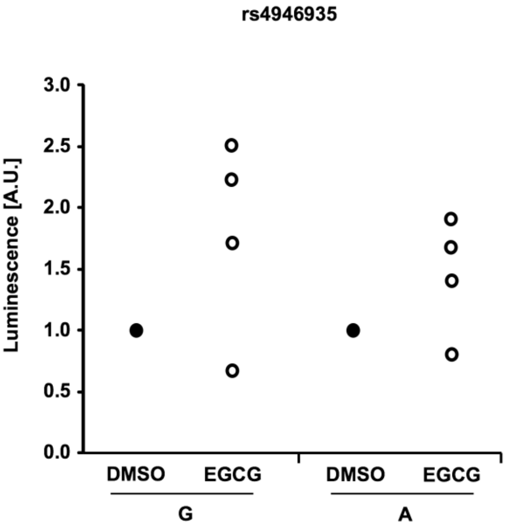

	Luminescence [A.U.] DMSO/50 µM EGCG	
	**rs4946935-G**	**rs4946935-A**
**Median**	1.96	1.55
**Min**	0.69	0.82
**Max**	2.52	1.91

7c

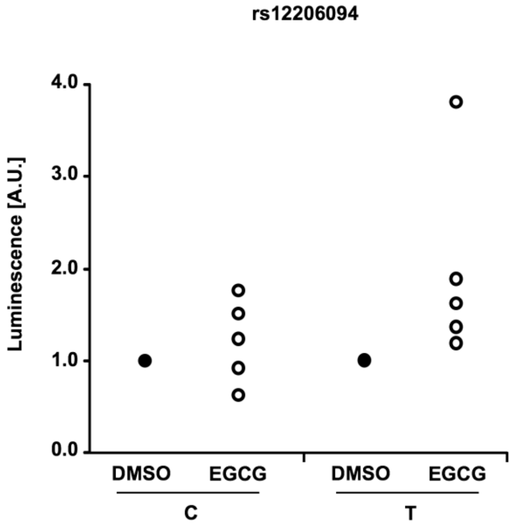

	Luminescence [A.U.] DMSO/50 µM EGCG	
	**rs12206094-C**	**rs12206094-T**
**Median**	1.25	1.63*
**Min**	0.64	1.20
**Max**	1.77	3.82

*, $P < 0.05$ *vs*. respective DMSO control (two-sided Wilcoxon signed-rank test for paired data)

7d

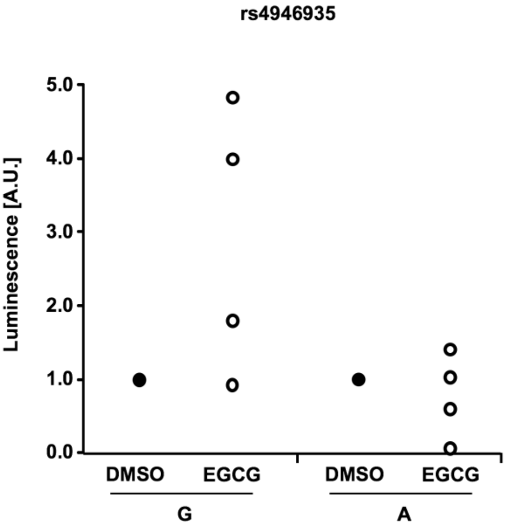

	Luminescence [A.U.] DMSO/50 µM EGCG	
	**rs4946935-G**	**rs4946935-A**
**Median**	2.91	0.83
**Min**	0.94	0.07
**Max**	4.84	1.42

Supplementary Figure 7. Effect of epigallocatechingallate (EGCG) on luciferase promoter activity in Panc1 **(7a, 7b)** and Jurkat **(7c, 7d)** cells. **(7a-7d)** In both cell lines, the activity of cells treated with DMSO instead of EGCG was set = 1 (black dot) for each allele of each SNV. Each white dot represents one independent experiment (n = 3 **(7a)** and n=4 **(7b)** for Panc1 cells and n = 5 **(7c)** and n = 4 **(7d)** for Jurkat cells). Final DMSO concentrations did not exceed 0.1%. The tables below the figures show the median as well as the minimum and maximum values for the ratios of the activity in presence of DMSO and 50 µM EGCG for each allele, taking into account all experiments. For determination of specific luciferase activity, activity of the firefly luciferase was normalized to the activity of the renilla luciferase. A.U., arbitrary luminescence units; EGCG, epigallocatechingallate.

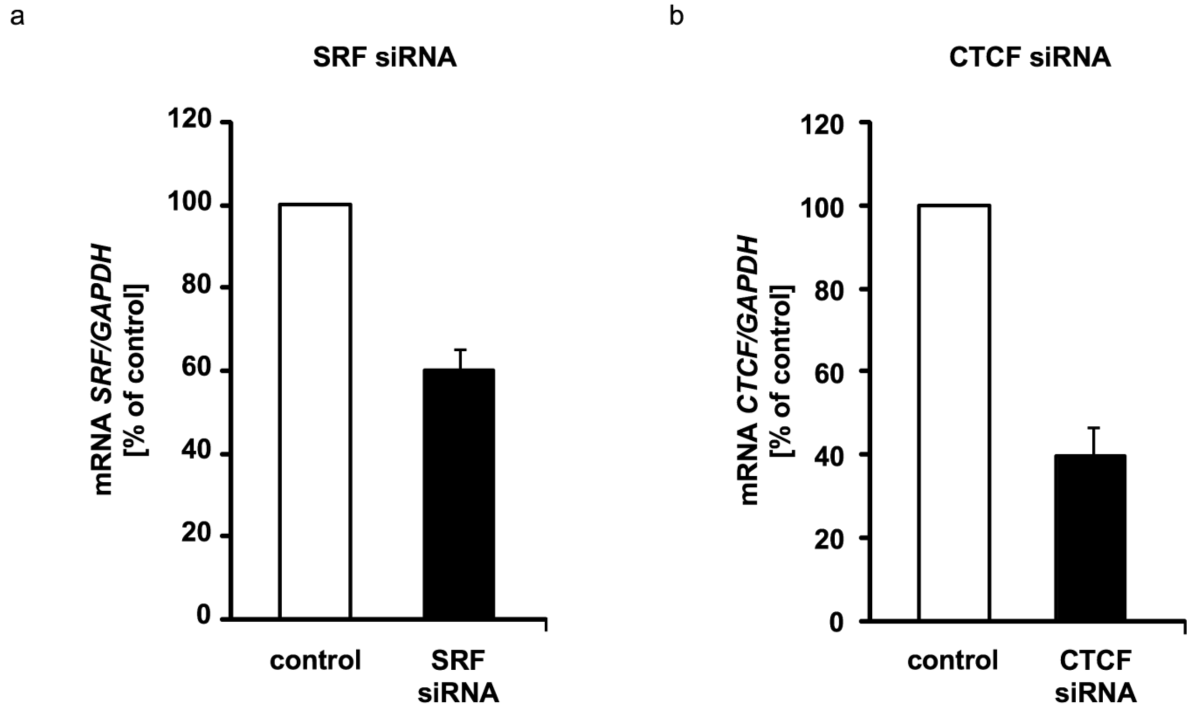

Supplementary Figure 8. Verification of transcription factor knockdown in Panc1 cells at the time of luciferase assay (six days after siRNA transfection). Shown is the mRNA expression of SRF **(a)** and CTCF **(b)** in the cells transfected with the transcription factor-specific siRNA in percent of control siRNA-transfected cells, whose expression was set 100%. Data are means + S.E.M; n=4 independent experiments; CTCF, CCCTC-binding factor; SRF, serum response factor; GAPDH, glyceraldehyde-3-phosphate dehydrogenase.

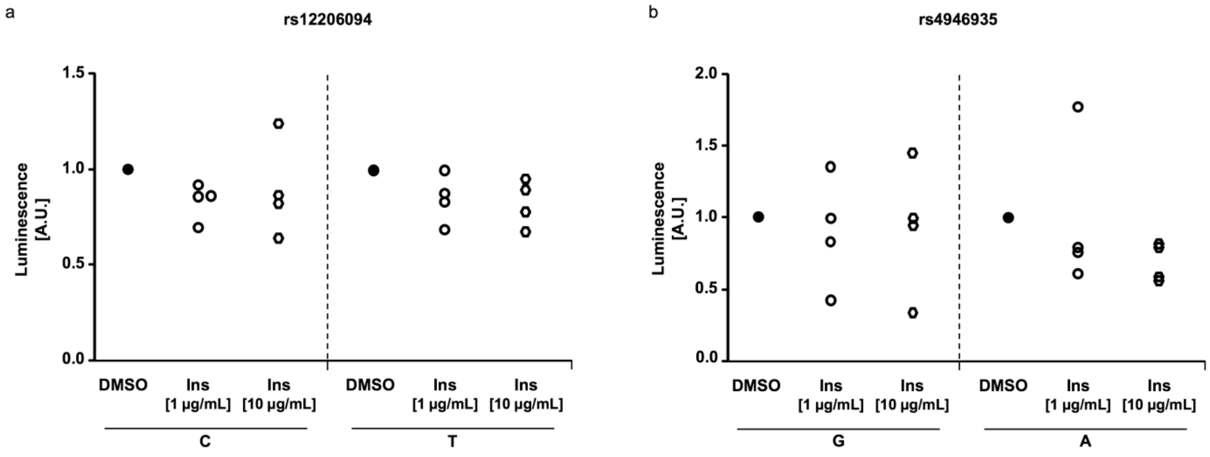

	Luminescence [A.U.] DMSO/Ins			
	1 µg/mL		10 µg/mL	
	C	T	C	T
**Median**	0.86[n.s.]	0.86	0.85	0.84[n.s.]
**Min**	0.70	0.69	0.65	0.67
**Max**	0.92	1.00	1.25	0.95

n.s., *P* = 0.068
(two-sided Wilcoxon signed-rank test for paired data)

	Luminescence [A.U.] DMSO/Ins			
	1 µg/mL		10 µg/mL	
	G	A	G	A
**Median**	0.92	0.78	0.97	0.70[n.s.]
**Min**	0.43	0.61	0.34	0.56
**Max**	1.36	1.78	1.46	0.82

n.s., *P* = 0.068
(two-sided Wilcoxon signed-rank test for paired data)

Supplementary Figure 9. Effect of 1 µg/mL and 10 µg/mL, respectively, insulin on luciferase promoter activity in Panc1 cells in presence of either allele of rs12206094 (**a**) and rs4946935 (**b**). (**a, b**) The activity of cells treated with DMSO instead of insulin was set = 1 (black dot) for each allele of each SNV. Each white dot represents one independent experiment (n = 4). Final DMSO concentrations did not exceed 0.1%. The duration of insulin treatment was 48 hours. The tables below the figures show the median as well as the minimum and maximum values for the ratios of the activity in presence of DMSO and 1 µg/mL or 10 µg/mL insulin, respectively, for each allele, taking into account all experiments. For determination of specific luciferase activity, activity of the firefly luciferase was normalized to the activity of the renilla luciferase. A.U., arbitrary luminescence units.

10a SNV rs12206094; SNV alleles: C/T; minor allele (= Homo Alt): T

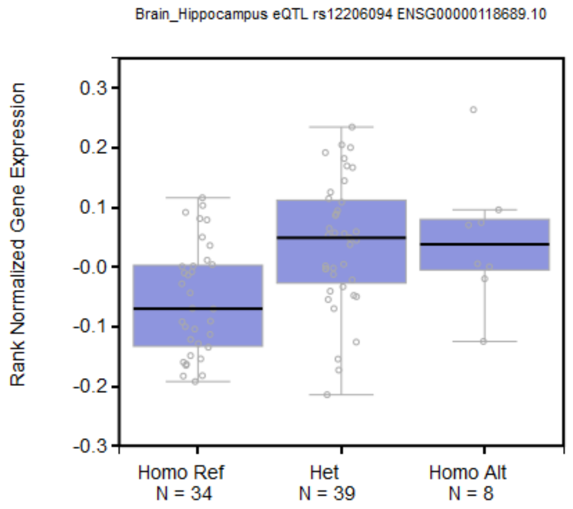

eQTL *P*-value = 4.1E-4

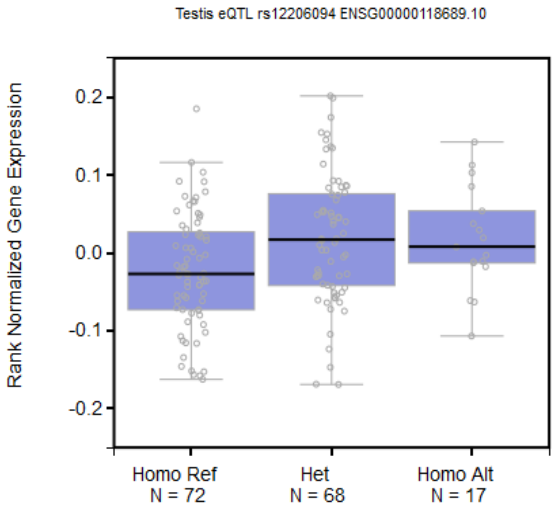

eQTL *P*-value = 0.0037

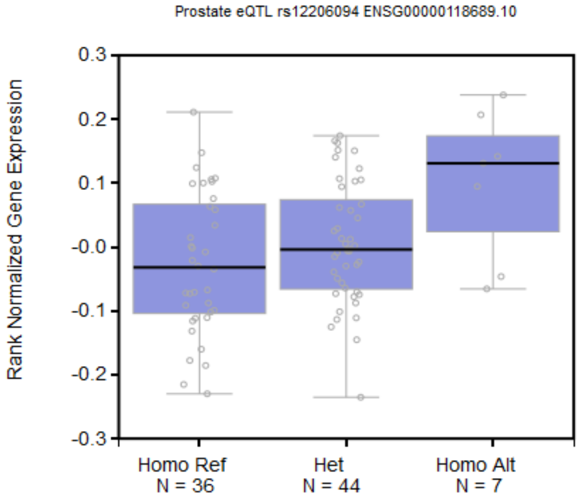

eQTL *P*-value = 0.007

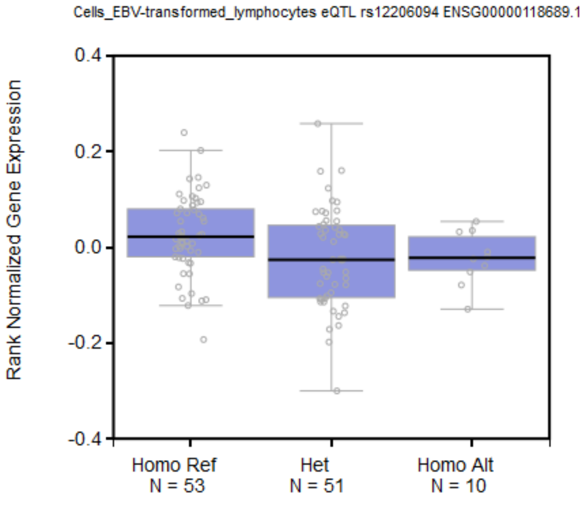

eQTL *P*-value = 0.01

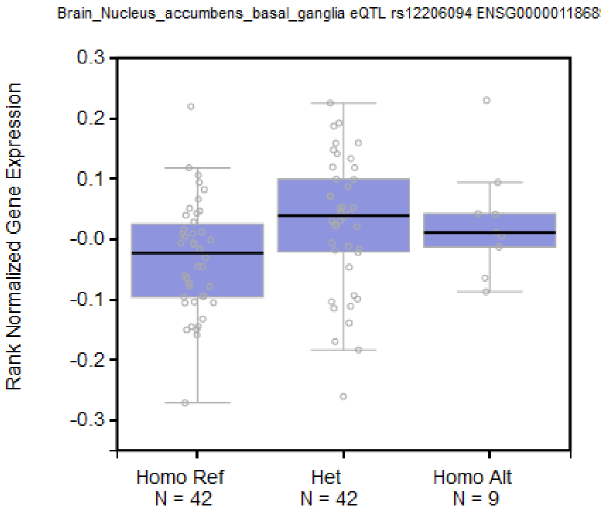

eQTL *P*-value = 0.013

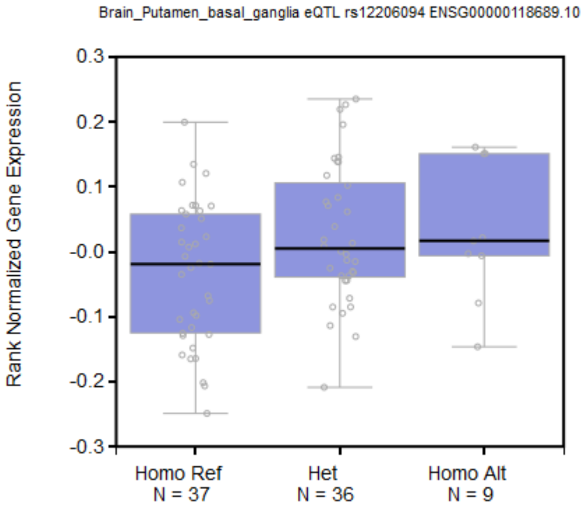

eQTL *P*-value = 0.023

10a *cont.*

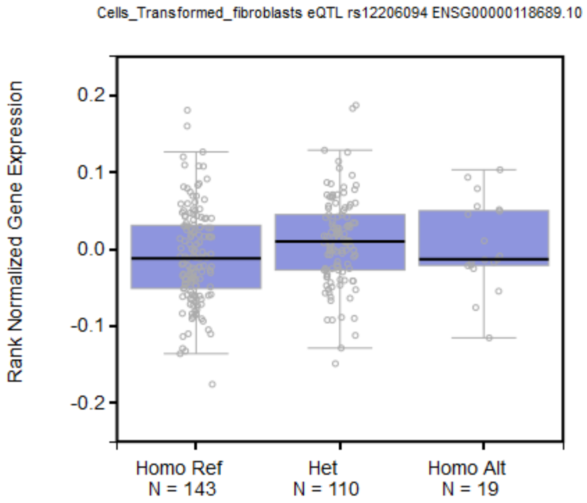

eQTL *P*-value = 0.024

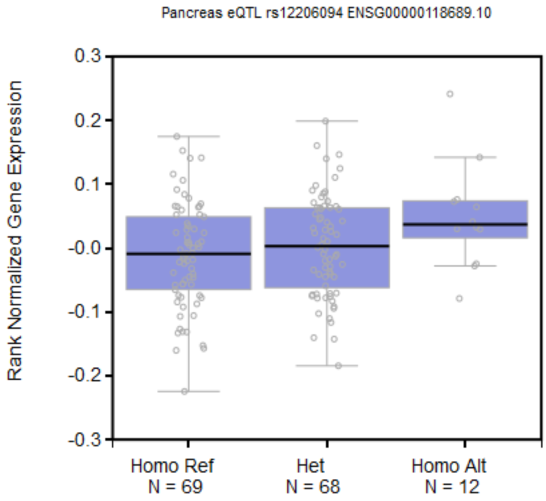

eQTL *P*-value = 0.026

10b SNV rs4946935; SNV alleles: A/G; minor allele (= Homo Ref): A

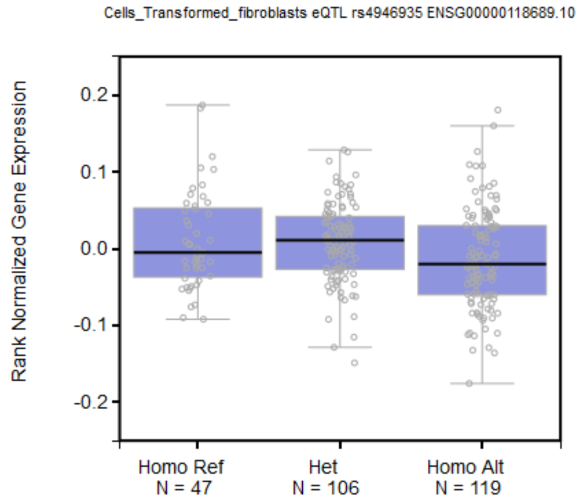

eQTL *P*-value = 0.002

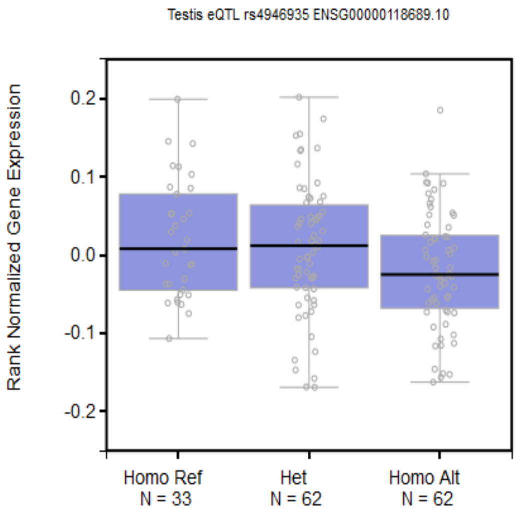

eQTL *P*-value = 0.0053

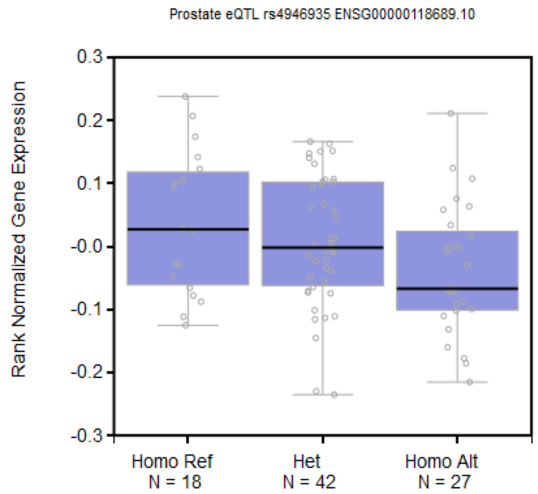

eQTL *P*-value = 0.0094

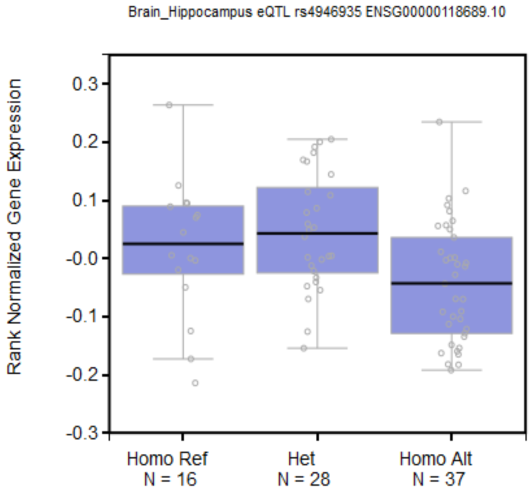

eQTL *P*-value = 0.0098

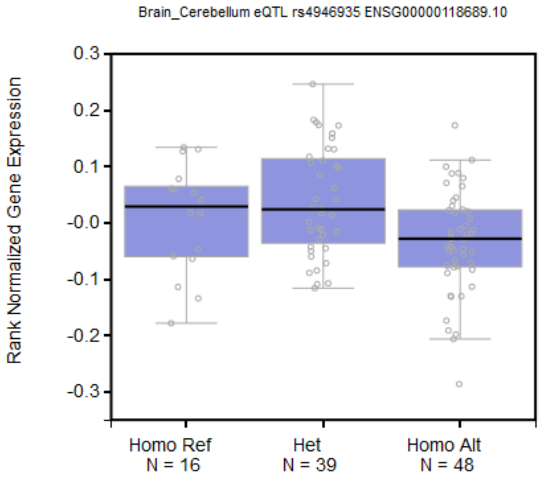

eQTL *P*-value = 0.012

Supplementary Figure 10. eQTL box plots for the two top-associated SNVs rs12206094 **(a)** or rs4946935 **(b)** and *FOXO3* in different tissues generated with the "Test Your Own SNP-Gene Associations" section (http://www.gtexportal.org/home/testyourown) from the GTEx database. Listed are plots which represent significant eQTL *P*-values. Homo, homozygous; Het, heterozygous; REF, reference allele as determined by the hg19/GRCh37 human genome reference; ALT, alleles that are alternate in comparison to the reference; eQTL, expression quantitative trait loci; eQTL *P*-value, nominal *P*-values were generated for each variant-gene pair by testing the alternative hypothesis that the slope of a linear regression model between genotype and expression deviates from 0.

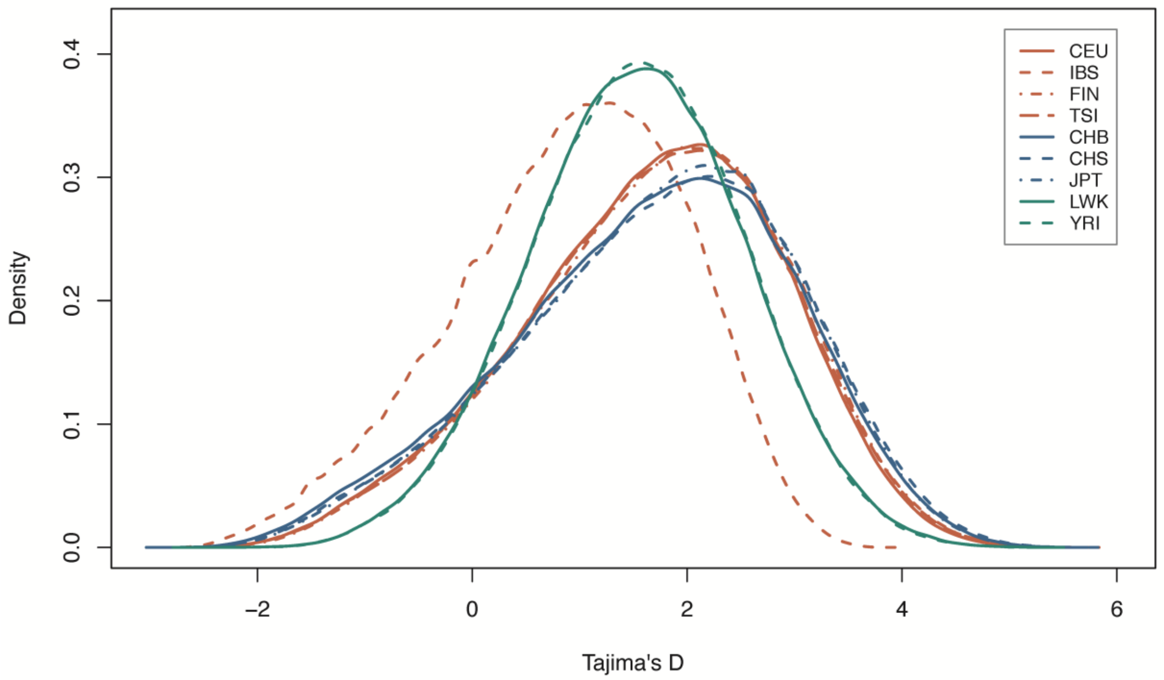

Supplementary Figure 11. Autosome-wide distribution of Tajima's D values in selected populations from the 1000 Genomes Pilot 1 data. Kernel density estimates (Gaussian kernel) for the distribution of Tajima's D values based on 255,393 autosomal regions in selected populations. CEU, Utah residents with ancestry from Northern and Western European, USA; IBS, Iberian Populations in Spain; FIN, Finnish in Finland; TSI, Toscani in Italia; CHB, Han Chinese in Beijing, China; CHS, Han Chinese South, China; JPT, Japanese in Tokyo, Japan; LWK, Luhya in Webuye, Kenya; YRI, Yoruba in Ibadan, Nigeria. Source: dbPSHP, http://jjwanglab.org/dbpshp[2].

Supplementary Table 1. Descriptions of the samples used in the three sequencing approaches, separately for each method, and number of cases and controls when all three sequencing methods are combined.

**Sample descriptives**	**Sequencing technology**			
	Sanger sequencing	SBS sequencing (Illumina)	SBL sequencing (SOLiD)	**combined**
**No. cases**	**138**	**48**	**34**	**118***
No. females	109	33	25	
No. males	29	15	9	
age range (years)	99-109	100-110	100-105	
**No. controls**	**92**	**46**	**22** (incl. 6 HapMap)	**158***
No. females	69	31	9	
No. males	23	15	7	
age range (years)	60-75	61-75	64-75	
**No. HapMap controls**			6	
females			3	
males			3	
age range (years)			NA	

*For quality control, there was an overlap of samples: one control sample overlapped between Sanger and SBS sequencing, one control sample overlapped between SBS and SBL sequencing, one case sample overlapped between Sanger and SBS sequencing, and one case sample overlapped between SBS and SBL sequencing. No., number; SBS, sequencing by synthesis; SBL, sequencing by ligation; NA, data not available.

Supplementary Table 2. Association between rs12206094 and rs4946935 and age-related phenotypes in all Danish LLI and controls.

	rs12206094						rs4946935					
	LLI			Controls			LLI			Controls		
**Age-related phenotype**	**N**	**OR**	***P***	**N**	**OR**	***P***	**N**	**OR**	***P***	**N**	**OR**	***P***
Chair stand	622	1.24	0.088	NA	NA	NA	623	1.16	0.221	NA	NA	NA
MMSE	626	0.99	0.935	NA	NA	NA	627	1.00	0.976	NA	NA	NA
**Age-related phenotype**	**N**	**β**	***P***	**N**	**β**	***P***	**N**	**β**	***P***	**N**	**β**	***P***
Age at first child	478	0.08	0.809	NA	NA	NA	478	0.21	0.536	NA	NA	NA
Age at last child	450	-1.01	**0.018**	NA	NA	NA	450	-0.76	0.074	NA	NA	NA
Age at menopause	NA	NA	NA	NA	NA	NA	NA	NA	NA	NA	NA	NA
Chair stand, timed	NA	NA	NA	720	0.40	**0.034**	NA	NA	NA	717	0.16	0.388
Cognitive composite score	562	-0.31	0.593	731	-0.95	0.117	563	-0.49	0.400	728	-0.62	0.290
Grip strength	559	0.19	0.566	728	-0.002	0.096	560	0.09	0.783	725	-0.14	0.698
Number of biological children	640	-0.17	0.122	709	0.01	0.843	641	-0.14	0.172	706	0.02	0.783

N, number; OR, odds ratio; *P*, *P*-value obtained from linear or ordinal logistic regression adjusted for age at assessment and gender. The *P*-values are not adjusted for multiple testing. *P*-values ≤ 0.05 are shown in bold. MMSE, Mini-Mental-State-Examination; NA, phenotype not available.

Supplementary Table 3. Association between rs12206094 and rs4946935 and age-related phenotypes in male Danish LLI and controls.

	rs12206094						rs4946935					
	LLI			Controls			LLI			Controls		
**Age-related phenotype**	**N**	**OR**	***P***	**N**	**OR**	***P***	**N**	**OR**	***P***	**N**	**OR**	***P***
Chair stand	145	1.01	0.984	NA	NA	NA	146	1.04	0.886	NA	NA	NA
MMSE	146	0.67	0.106	NA	NA	NA	147	0.70	0.145	NA	NA	NA
**Age-related phenotype**	**N**	**β**	***P***	**N**	**β**	***P***	**N**	**β**	***P***	**N**	**β**	***P***
Age at first child	73	0.23	0.799	NA	NA	NA	73	0.10	0.906	NA	NA	NA
Age at last child	72	-1.07	0.260	NA	NA	NA	72	-1.04	0.271	NA	NA	NA
Age at menopause	NA	NA	NA	NA	NA	NA	NA	NA	NA	NA	NA	NA
Chair stand, timed	NA	NA	NA	283	0.47	0.072	NA	NA	NA	282	0.01	0.979
Cognitive composite score	134	-0.94	0.470	285	-0.39	0.690	135	-0.82	0.499	284	1.22	0.195
Grip strength	132	0.34	0.712	285	-0.13	0.860	133	0.86	0.323	284	-0.09	0.904
Number of biological children	149	0.02	0.934	273	0.02	0.887	150	0.27	0.238	272	0.03	0.748

N, number; OR, odds ratio; *P*, *P*-value obtained from linear or ordinal logistic regression adjusted for age at assessment and gender. The *P*-values are not adjusted for multiple testing. *P*-values ≤ 0.05 are shown in bold. MMSE, Mini-Mental-State-Examination; NA, phenotype not available.

Supplementary Table 4. Association between rs12206094 and rs4946935 and age-related phenotypes in female Danish LLI and controls.

	rs12206094						rs4946935					
	LLI			Controls			LLI			Controls		
**Age-related phenotype**	**N**	**OR**	***P***	**N**	**OR**	***P***	**N**	**OR**	***P***	**N**	**OR**	***P***
Chair stand	477	1.33	**0.047**	NA	NA	NA	477	1.21	0.172	NA	NA	NA
MMSE	480	1.10	0.457	NA	NA	NA	480	1.11	0.421	NA	NA	NA
**Age-related phenotype**	**N**	**β**	***P***	**N**	**β**	***P***	**N**	**β**	***P***	**N**	**β**	***P***
Age at first child	405	0.07	0.850	NA	NA	NA	405	0.24	0.529	NA	NA	NA
Age at last child	378	-1.01	**0.033**	NA	NA	NA	378	-0.72	0.132	NA	NA	NA
Age at menopause	346	-0.11	0.771	433	-0.78	0.087	346	0.39	0.300	431	-0.87	**0.047**
Chair stand, timed	NA	NA	NA	437	0.36	0.174	NA	NA	NA	435	0.24	0.347
Cognitive composite score	428	-0.13	0.846	446	-1.24	0.111	428	-0.37	0.574	444	-1.62	**0.031**
Grip strength	427	0.14	0.673	443	0.06	0.878	427	-0.15	0.636	441	-0.17	0.647
Number of biological children	491	-0.22	0.072	436	0.01	0.908	491	-0.26	**0.027**	434	0.01	0.928

OR, odds ratio; *P*, *P*-value obtained from linear or ordinal logistic regression adjusted for age at assessment and gender. The *P*-values are not adjusted for multiple testing. *P*-values ≤ 0.05 are shown in bold. MMSE, Mini-Mental-State-Examination; NA, phenotype not available.

Supplementary Table 5. Descriptives of age-related phenotypes investigated in the Danish LLI and controls.

	LLI		Controls	
**Age-related phenotype**	**N**	**No. individuals (%)**	**N**	**No. individuals (%)**
Chair stand				
Cannot	623	52 (8.3%)	NA	NA
Can, with use of arms		256 (41.1%)		NA
Can, without use of arms		315 (50.6%)		NA
MMSE				
Severe impairment (0-17)	627	110 (17.6%)	NA	NA
Mild impairment (18-22)		153 (24.4%)		NA
Normal (23-27)		246 (39.2%)		NA
Maximum (28-30)		118 (18.8%)		NA
**Age-related phenotype**	**N**	**Mean (SE)**	**N**	**Mean (SE)**
Age at first child	478	26.99 (0.23)	NA	NA
Age at last child	450	34.35 (0.28)	NA	NA
Age at menopause	346	49.22 (0.25)	433	48.96 (0.28)
Chair stand, timed	NA	NA	721	9.71 (0.12)
Cognitive composite score	563	27.63 (0.38)	732	46.99 (0.37)
Grip strength	560	15.96 (0.27)	729	36.49 (0.43)
Number of biological children	641	2.40 (0.07)	709	2.07 (0.04)

MMSE, Mini-Mental-State-Examination; N, total number of individuals; NA, phenotype not available; SE, standard error.

Data on age-related phenotypes was collected as part of a comprehensive home-based interview focusing on health and lifestyle issues as well as objective assessments of cognitive and physical abilities. Cognitive function was assessed by the MMSE and a cognitive composite score, which evaluates verbal fluency, forward and backward digit span, and immediate and delayed recall[3]. The MMSE score ranges from 0 to 30 and was here divided into four groups: severe impairment (MMSE 0-17), mild impairment (MMSE 18-22), normal impairment (MMSE 23-27), and maximum impairment (28-30). The cognitive composite score was standardized using the mean and standard deviation of cognitive composite scores measured in 46-50-year olds born from 1949-1952. Physical function was assessed by chair stand and grip strength. In the younger controls, chair stand was measured as the time used (in seconds) to stand up from a chair five times in a row as quickly as possible, whereas in the LLI chair stand assessed the ability to stand up from a chair with the outcome categories ‘cannot’, ‘can, with use of arms’, and ‘can, without use of arms’. Grip strength was measured using a handheld dynamometer (SMEDLEY’s dynamometer, Scandidact, Kvistgaard, Denmark) and the maximum of three measurements with the strongest hand was used. Information about age at first child, age at last child, age at menopause and number of biological children was obtained by asking the questions: ‘How old were you when you had your first/last child?’, ‘At what age did you reach menopause?’, and ‘How many biological children do you have?’.

Supplementary Table 6. Prediction of CCCTC-binding factor (CTCF) binding sites in *FOXO3* sequences containing either rs12206094-C or rs12206094-T.

Motif PWM	Motif sequence	Motif start location	Motif length	Motif orientation	PWM score
Input sequence: GRCh38:6:108584752:108585151:1, rs12206094-C					
EMBL_M1	CACCCTGTGCAGGC	165	14	-	7.20
EMBL_M2	AGAATTGCT	250	9	+	7.84
REN_20	GTAGCCTGCACAGGGTGGTA	162	20	+	9.59
MIT_LM2	CCTGCACAGGGTGGTAGAG	166	19	+	3.99
MIT_LM7	TAGCCTGCACAGGGTGGTAG	163	20	+	6.38
**MIT_LM23**	**TTGCCACCAAGAATTGCTAT**	**241**	20	**+**	**5.29**
Input sequence: GRCh38:6:108584752:108585151:1, rs12206094-T					
EMBL_M1	CACCCTGTGCAGGC	165	14	-	7.28
EMBL_M2	AGAATTGCT	250	9	+	7.83
REN_20	GTAGCCTGCACAGGGTGGTA	162	20	+	9.61
MIT_LM2	CCTGCACAGGGTGGTAGAG	166	19	+	4.03
MIT_LM7	TAGCCTGCACAGGGTGGTAG	163	20	+	6.41
**MIT_LM23**	**TAGCCTGCACAGGGTGGTAG**	**163**	20	**+**	**2.23**

The CCCTC-binding factor (CTCF) binding site database (CTCFBSDB 2.0; http://insulatordb.uthsc.edu/home_new.php)[4,5] for CTCF binding site identification uses a position weight matrix (PWM) of six core motifs of CTCF binding sites. These core motifs include the EMBL_M1 and EMBL_M2 motifs[6], the Ren_20 motif[7], and the LM2, LM7 and LM23 motifs[8]. STORM[9] program is used in the CTCFBSDB to scan for each PWM in the query sequences and a score is calculated based on the log-odds of the observed sequence being generated by the motif versus being generated by the background. Usually, a PWM score >3.0 is a suggestive match[5]. For more information, please refer to the CTCFBSDB 2.0 webpage (http://insulatordb.uthsc.edu/home_new.php). The *FOXO3* sequence containing rs12206094-C was predicted to comprise a LM23-similar CTCFBS motif with a score of 5.29; on the contrary, in the presence of rs12206094-T, a much lower score of only 2.23 was detected. In the table, the lines referring to the LM23 motif are highlighted in bold.

Supplementary Table 7. Results of significant eQTL associations for rs12206094 and rs4946935 (and SNVs in LD with these two).

SNV	SNV position (hg18)	eQTL *P*-value	Tissue	Chr.	SNV alleles	minA	Effect direction of minA	eQTL gene	eQTL gene position (hg18)	Browser
rs12206094	109012893	4.1E-4	brain - hippocampus	6	C/T	T	+	FOXO3	chr6:108988762-109112664	GTEx (Test Your Own)*
		0.0037	testis				+			
		0.0070	prostate				+			
		0.010	cells - EBV-transformed lymphocytes				-			
		0.013	brain - nucleus accumbens (basal ganglia)				+			
		0.023	brain - putamen (basal ganglia)				+			
		0.024	cells - transformed fibroblasts				+			
		0.026	pancreas				+			
rs4946935	109107435	0.0020	cells - transformed fibroblasts	6	A/G	A	+	FOXO3	chr6:108988762-109112664	GTEx (Test Your Own)*
		0.0053	testis				+			
		0.0094	prostate				+			
		0.0098	brain - hippocampus				+			
		0.012	brain - cerebellum				+			
rs1536057**	108992316	6.9E-5	nerve - tibial	6	C/T	T	-	LINC00222	chr6:109179550-109197838	GTEx
		4.3E-4	lung				-			
		4.5E-4	thyroid				-			
		0.009	brain-cerebellum				-			
rs2022464***	109052063	3.7E-5	nerve - tibial	6	A/C	A	-	LINC00222	chr6:109179550-109197838	GTEx
		5.2E-5	thyroid				-			
		10.0E-3	lung				-			
		4.4E-3	brain-cerebellum				-			
		0.006	adrenal gland				-			
		0.007	artery tibial				-			

Supplementary Table 7 *cont.*

SNV	SNV position (hg18)	eQTL *P*-value	Tissue	Chr.	SNV alleles	minA	Effect direction of minA	eQTL gene	eQTL gene position (hg18)	Browser
rs2153960****	109094877	7.6E-5	thyroid	6	C/T	C	-	LINC00222	chr6:109179550-109197838	GTEx
		7.3E-4	nerve - tibial				-			
		3.4E-3	adipose-visceral				-			
		0.007	muscle-skeletal				-			
rs4946935	109107435	2.3E-6	whole blood	6	A/G	A	-	HS.133419	chr6:109128786-109129470	Blood eQTL browser
rs12206094	109012893	3.1E-4	whole blood	6	C/T	T	-	HS.133419	chr6:109128786-109129470	Blood eQTL browser

*the corresponding eQTL box plots are listed in Supplementary Fig. 10

**in LD with rs12206094; r^2 = 1.00 based on HapMap-CEU individuals, 1000 Genomes

***in LD with rs4946935; r^2 = 0.96 based on HapMap-CEU individuals, 1000 Genomes

****in LD with rs4946935; r^2 = 0.96 based on HapMap-CEU individuals, 1000 Genomes

SNV, single nucleotide variant; Chr, chromosome; minA, minor allele; eQTL, expression quantitative trait loci

eQTL *P*-value, GTEx information: nominal *P*-values were generated for each variant-gene pair by testing the alternative hypothesis that the slope of a linear regression model between genotype and expression deviates from 0

GTEx (Test Your Own SNP-Gene Associations): http://www.gtexportal.org/home/testyourown

GTEx: http://www.gtexportal.org/home/

Blood eQTL browser: http://genenetwork.nl/bloodeqtlbrowser/[10]

Supplementary Table 8. Allele frequencies for the two *FOXO3* SNVs rs4946935 and rs12206094 across diverse populations according to NCBI dbSNV (accessed September 07, 2016) and in the control group of the present study (in italics).

**Ancestry**	**Population**	**Individual group** (Origin, residency)	**rs4946935** Sample size	**rs4946935** Allele frequencies A*, #	**rs4946935** Allele frequencies G	**rs12206094** Sample size	**rs12206094** Allele frequencies C#	**rs12206094** Allele frequencies T*
**European**	HapMap-CEU	Europeans, US	118	0.297	0.703	226	0.726	0.274
			226	0.265	0.735			
	HapMap-TSI	Tuscans, Italy	176	0.341	0.659	176	0.648	0.352
	Controls in our study	*Germans, Germany*	*918*	*0.304*	*0.696*	*918*	*0.713*	*0.287*
**Asian**	HapMap-HCB	Han Chinese, China	90	0.178	0.822	86	0.860	0.140
			86	0.174	0.826			
	HapMap-CHB	Han Chinese, China	82	0.171	0.829	82	0.854	0.146
	HapMap-JPT	Asians, Japan	90	0.189	0.811	172	0.843	0.157
			172	0.192	0.808			
**African**	HapMap-YRI	Yorubas, Nigeria	120	0.983	0.017	226	0.558	0.442
			226	0.951	0.049			
	HapMap-LWK	Luhyas, Kenya	180	0.822	0.178	180	0.672	0.328
	HapMap-MKK	Maasai, Kenya	286	0.636	0.364	284	0.676	0.323
**Ancient DNA**[b]		Hunter-gatherers	9	0.620[a]	0.380[a]	9	0.463	0.537
		Early farmers	79	0.453[a]	0.547[a]	79	0.525	0.475

*, longevity allele,

#, ancestral allele,

[a], LD-SNP (rs1935949) of rs4946935, r^2 (CEU) = 0.96 (1000 genomes pilot 1; SNAP Proxy Search; https://archive.broadinstitute.org/mpg/snap/ldsearch.php).

[b]Data from Mathieson et al. (2015)[11]

Supplementary Table 9. Allele frequencies and Tajima's D values in selected populations from the 1000 Genomes Pilot 1 data.

	EUR				EAS			AFR	
	**CEU**	**IBS**	**FIN**	**TSI**	**CHB**	**CHS**	**JPT**	**LWK**	**YRI**
**rs12206094**									
**DAF**	0.26	0.32	0.26	0.35	0.14	0.22	0.16	0.31	0.45
**Tajima's D**	1.94 (0.57)	0.96 (0.50)	1.98 (0.57)	2.09 (0.61)	-0.20 (0.09)	0.37 (0.19)	0.10 (0.15)	1.39 (0.44)	1.29 (0.40)
**rs4946935**									
**DAF**	0.75	0.71	0.65	0.66	0.82	0.74	0.81	0.19	0.05
**Tajima's D**	2.52 (0.75)	1.61 (0.73)	2.50 (0.73)	3.57 (0.95)	0.42 (0.20)	1.58 (0.45)	0.95 (0.29)	1.56 (0.51)	0.91 (0.27)

Given are absolute values and, in parentheses, the corresponding quantile with regard to the autosome-wide distribution of those values in selected populations. Quantiles are within the main body of the population-specific distributions, except for Tuscans, where the observed value was within the top 5% of all autosomal values. DAF, derived allele frequency; EUR, European meta-population; EAS, East Asian meta-population; AFR, African meta-population; CEU, Utah residents with ancestry from Northern and Western European, USA; IBS, Iberian populations in Spain; FIN, Finnish in Finland; TSI, Toscani in Italy; CHB, Han Chinese in Beijing, China; CHS, Han Chinese South, China; JPT, Japanese in Tokyo, Japan; LWK, Luhya in Webuye, Kenya; YRI, Yoruba in Ibadan, Nigeria. Source: dbPSHP, http://jjwanglab.org/dbpshp[2].

Supplementary Table 10. Sequences of primers used for *FOXO3* sequencing with the SOLiD sequencing by ligation technology.

Primer name	Sequence 5‘-3‘	Amplicon
SFOXO3A_1E_F	TTGAGAGATACATGAAGCACAGTAAGT	7467 bp
SFOXO3A_1E_R	GGAATTCTACCCTAATAGAGAATGGAC	
FX3A_2.f	GGTCTCATCCCTGAGGTGAA	6976 bp
FX3A_2.r	ATGACCCTGGTTGATGGTGT	
FX3A_3_A1_F1	GGAAGGGAGGTAAGCCTTTCTA	3960 bp
FX3A_3_A1_R1	CACTTCCCCAACCTGTCTAAAG	
**gap in long-range PCR production**		
FX3A_4_A2_F5	TGTAATGACTTCCTGCAGTTGG	4411 bp
FX3A_4_A2_R3	CCCAGCAACTTCTTAGCAGAGT	
FX3A_5_A1_F3	ATTTCACAAGTGTGGGGGATAC	6033 bp
FX3A_5_A1_R2	ATCCTTGGTGTTCTTTGGCTTA	
FX3A_5_A2_F1	CAACCAGCCTTCCTTGTATTTC	4930 bp
FX3A_5_A2_R5	ATCGCAACGCTTTTAAATTAGG	
FX3A_6_F2	AAAGGGACAACTCTGGGTACAA	9228 bp
FX3A_6_R1	TCAGCAAGACTTCCCTTCTTTC	
**gap in long-range PCR production**		
FX3A_7_A2_F6	AAAGGAACGTGCTTCCTAAGTG	5121 bp
FX3A_7_A2_R6	CAAGTCCAGCTAACATGATCCA	
FX3A_8.f	GTTGGTGCACGTGGTAATTG	7278 bp
FX3A_8.r	GTCAGCCACAGAAGCTTTCC	
FX3A_9_F1	CACCTGCATCTGTGTTGGTC	9428 bp
FX3A_9_R2	AGCAGACAATGCCCTCAGTC	
**gap in long-range PCR production**		
FX3A_11.f	CAGGATGAGCATGCCACTAA	9043 bp
FX3A_11.r	CATGGCCAACTAGGGAACAT	
FX3A_12.f	TTTCCTAGCAAGTGGACGCT	7545 bp
FX3A_12.r	CTGAAGGTTGCCTGTGGATT	
FX3A_13.f	AGCCAAGCTTTGATGCATTT	8455 bp
FX3A_13.r	TTTGTGCTTCAAGTCTCATGC	
FX3A_14.f	CAAGCCTGTGGTGTATGTGG	9145 bp
FX3A_14.r	CGACACTCCTGGGTGCTAAT	
FX3A_15_F1	TCCAGAAAGTTTGGGGAGATAA	7869 bp
FX3A_15_R1	GAATGCTGTGTTTGGTTGAAAA	
FX3A_16.f	CCTTAAGGAAGGCACATCCC	7790 bp
FX3A_16.r	AAGTACGACCCAAGCTTCCC	
**gap in long-range PCR production**		
FX3A_17_A2_F1	TGTGAAATGGACCTGGTGAA	5591 bp
FX3A_17_A2_R1	CCATGCTTTAAGGCCCACTA	
FX3A_18_F5	CCCAGTAAATGCAGTGAAGAGTC	9378 bp
FX3A_18_R5	CTCCCATTAACAGGAGTGACAAG	
FX3A_19.f	CAGGGCTCCAGAACCTGATA	6725 bp
FX3A_19.r	AGCTCTGCTCTGAAAGCTGG	
FX3A_20_A1_F1	GGTGATTTCCACACACAGAAGA	4509 bp
FX3A_20_A1_R1	TACGGTTCTTTCCTTTTCCTCA	
FX3A_20_A2n_F1	ATTCATGCACTGGATGTGGA	5807 bp
FX3A_20_A2n_R1	TCATCAAAACTGCTGCGAAC	
FX3A_21.f	TTTCCACATTGTGCCTACCA	2637 bp
FX3A_21.r	AGGCAGCCTCAGGAGTGTTA	

Supplementary Table 11. Sequences of primers used for *FOXO3* sanger sequencing and long-range PCR.

Gene region	Primer name	Sequence (5´-3´)	Amplicon
***Long-range* PCR**			
Promoter, Exon1, Exon2	F3_LR_34_MM2_F	GGCAGATTTGGCCAGATAAC	8407 bp
	F3_LR_34_M02_R	CCCAACTGCAGGAAGTCATT	
Exon3	FOX_ex3_LR_2F	AAAGGTAGACGTTGTGCCTCTATACTA	2704 bp
	FOX_ex3_LR_2R	ACATGTAACACTGAAGATTGACAAAAG	
Exon4	FOXO3A_3U_LR1F	CCATTCCCTTTGGGCTTTTC	5523 bp
	FOXO3A_3U_LR1R	GGCCGTCTCTAAGTCCCAAC	
***Nested* PCR**			
Promoter, NM201559	ProFOX_201_F1	CCCTTTTTCCCTCTCCTCTC	690 bp
	ProFOX_201_R1	TTACCCCCGTCCTACATTCA	
	ProFOX_201_F2	CGCGAAACTCTCAATCAGGT	722 bp
	ProFOX_201_R2	GCCGCTTACTCGTCAAGG	
Promoter, NM001455	ProFOX_0014_F1	TGATGAACGTGCTGGTCCGGGT	766 bp
	ProFOX_0014_R1	CAGGAACAGGAGGACCTGAA	
	ProFOX_0014_F2	GGAGGAGGAATGTGGAAGGT	526 bp
	ProFOX_0014_R2	GGGTTTTGTGTGGTTTGCAT	
	ProFOX_0014_F3	TCTAACAGGGAACCGGACAC	729 bp
	ProFOX_0014_R3	CTCGCTTCCTTCCCTTCAG	
	ProFOX_0014_F4	GGGCACGGATCGTAGAATAA	602 bp
	ProFOX_0014_R4M_1R	AGGGAGAAGGGGGAGCGG	
Exon1	FOX_ex1_F	CTAGGTTGAGGCGCCCTG	386 bp
	FOX_ex1_R	TTAAAAACACTAGCGGGCGA	
Exon2	FOX_ex2_a1_F1	AACATAAACAAACGCACGCA	633 bp
	FOX_ex2_a1_R1	AGGGGCCACGTACAGGAT	
	FOX_ex2_a2_F4	GATGGCAGAGGCACCGGCT	702 bp
	FOX_ex2_a2_R2	ACTCCGACGAATCCGAGAC	
Exon3	FOX_ex3_a1_F1	CTATATCATCTGGGTGCTCGG	600 bp
	FOX_ex3_a1_R1	TTCAGTCAGCCCATCATTCA	
	FOX_ex3_a2_F2	CCATGCTCTACAGCAGCTCA	653 bp
	FOX_ex3_a2_R2	GGACTCACTCAAGCCCATGT	
	FOX_ex3_a3_F3	ATCCGATGATGTCCTTTGCT	636 bp
	FOX_ex3_a3_R3	ACCACTGCTCCATGGTTTTC	
Exon4	FOXO3A_ex4_F001	GATGGAAGGCCTTGACAGGT	529 bp
	FOXO3A_ex4_R001	AGGGGAAGGACGGTTAACAT	
	FOXO3A_ex4_F01	CGATGGTTTATGGGACGTTT	571 bp
	FOXO3A_ex4_R01	CATTTGGCAATGAGTGGAGA	
	FOXO3A_ex4_P_F1	TTGCTTTGCAGAACAAATGAA	414 bp
	FOXO3A_ex4_R1	CAAAGGTGGTCCCAACTATTCC	
	FOXO3A_ex4_F2	CTTTTTTTTCTGCTTCTATGGATTTC	606 bp
	FOXO3A_ex4_R2	TTCAGTAAAAGGCAGGGTGAA	

Supplementary Table 11, *cont.*

Gene region	Primer name	Sequence (5´-3´)	Amplicon
Exon4, *cont.*	FOXO3A_ex4_F3	TTGTGCGCCTTGGCTTTA	601 bp
	FOXO3A_ex4_R3	TTTTGTTAGTCACTTTGCATGTTTC	
	FOXO3A_ex4_F4	GGATGCATTGCAGAGGCACTA	608 bp
	FOXO3A_ex4_R4	CGGGACCCTAGACAGGCTTC	
	FOXO3A_ex4_F5	TGAAGAGGGAATGCTTTGGTT	603 bp
	FOXO3A_ex4_R5R	GGTATCAGGTTCTGGAGCCC	
	FOXO3A_ex4_F6	CAGCTGTAATGTTTGATTTATGATGA	662 bp
	FOXO3A_ex4_R6	TGGAGGACTTCTTTTTGACTGC	
	FOXO3A_ex4_F7	TGATTTCAGGTGGCTTCCAAA	669 bp
	FOXO3A_ex4_R7	GCATCCGCTTCAAGACCTCA	
	FOXO3A_ex4_F8	TATTTGGGTGAACATTGTATGATTAGG	652 bp
	FOXO3A_ex4_R8	AGATTATATGGGATATGAGCAAGGA	
	FOXO3A_ex4_F9	TGAAGGAGGACCAGAAAAATTAGTTAA	651 bp
	FOXO3A_ex4_R9	AGAGTCTCTCAGCTTTGTGGC	
	FOXO3A_ex4_F1La	CAAGTCTACGGGTGCCAGAT	358 bp
	FOXO3A_ex4_R1La	GCAAGGCTGAAAATAATCAAGG	

Supplementary Table 12. *In silico* analysis results for the SNVs detected in the coding exons 2 and 3.

SNV No.	SNV ID	Localization	AA Subsitiution	PhyloP[12]	Grantham Score[13]	PolyPhen-2[14]	SNPs&GO[15]	MutPred[16]	SIFT[17]	Mutation Taster2[18]	Mutation Assessor[19]	FATHMM[20]	IMHOTEP (RF)[21]
148	rs11757217	Exon 2	synonym	-	-	-	-	-	-	-	-	-	-
149	snv_chr6_108989376	Exon 2	G91A	consequential	inconsequential	inconsequential	inconsequential	inconsequential	inconsequential	consequential	inconsequential	consequential	consequential
150	rs111556510	Exon 2	A140V	consequential	inconsequential	inconsequential	inconsequential	inconsequential	inconsequential	inconsequential	inconsequential	consequential	inconsequential
151	rs150320900	Exon 2	synonym	-	-	-	-	-	-	-	-	-	-
769	rs61756661	Exon 3	synonym	-	-	-	-	-	-	-	-	-	-
770	rs145259784	Exon 3	A341T	inconsequential	inconsequential	inconsequential	consequential	inconsequential	inconsequential	inconsequential	inconsequential	consequential	consequential
771	rs374860833	Exon 3	synonym	-	-	-	-	-	-	-	-	-	-
772	rs181686373	Exon 3	A521S	consequential	inconsequential	inconsequential	inconsequential	inconsequential	inconsequential	consequential	inconsequential	inconsequential	consequential
773	snv_chr6_109092246	Exon 3	R506H	inconsequential	inconsequential	consequential	consequential	inconsequential	consequential	consequential	consequential	consequential	consequential
774	snv_chr6_109092372	Exon 3	R548H	inconsequential	inconsequential	inconsequential	inconsequential	inconsequential	inconsequential	consequential	inconsequential	consequential	consequential
775	rs113367269	Exon 3	synonym	-	-	-	-	-	-	-	-	-	-

SNV, single nucleotide variant; AA, amino acid; RF, random forest; -, no prediction (synonymous SNV)

Supplementary Table 13. *In silico* analysis results (MutationTaster2) for the associated SNVs detected in non-coding exon 4.

SNV No.	SNV ID	Prediction	Probability
946	rs4945816	inconsequential	1.000000
952	rs4946936	inconsequential	0.999977
956	rs9400241	inconsequential	0.999999
975	rs1062034	inconsequential	0.000185

SNV, single nucleotide variant

Supplementary Table 14. Sequences of primers and oligonucleotides used in the functional experiments.

**A. Sequences of primers used in the generation of the plasmids containing the specific *FOXO3* alleles of rs4946935 and rs12206094**	
rs4946935-G	Forward:<br>TAAT**G**TGGCTTTCTTTATCTCCCAAAGTGAATGATCCCATTCCCTTTGGGCTTT TCAACTTCAGAGCACGGGCTCGAGATCTGCGA<br>Reverse:<br>GGGGTGTTTTGGTGGGTCCTTTTTAGTTAGATGTTCAAGGTGCAGATAAGATT CTGTTCTAGACTTGGCTAGCACGCGTAAGAG
rs4946935-A	Forward:<br>TAAT**A**TGGCTTTCTTTATCTCCCAAAGTGAATGATCCCATTCCCTTTGGGCTTT TCAACTTCAGAGCACGGGCTCGAGATCTGCGA<br>Reverse:<br>GGGGTGTTTTGGTGGGTCCTTTTTAGTTAGATGTTCAAGGTGCAGATAAGATT CTGTTCTAGACTTGGCTAGCACGCGTAAGAG
rs12206094-T	Forward:<br>GGCACTTGCTTTGC**T**ACCAAGAATTGCTATCTCCAAAATCCGGGCTCGAGATC TGCGA<br>Reverse:<br>AAAGCCATAGATCAGGAATTCAACTTCTCATTTCAAAACGGGCTAGCACGCG TAAGAG
rs12206094-C	Primer used to generate the rs12206094-C plasmid from the rs12206094-T plasmid:<br>TTG GCA CTT GCT TTG C**C**A CCA AGA ATT GCT ATC TCC
**B. Oligonucleotides used in gel-shift experiments**	
rs4946935-A<br>rs4946935-G	5´- ACACCCCTAATATGGCTTTCTTT-3´<br>5´- ACACCCCTAATGTGGCTTTCTTT-3´
rs12206094-C<br>rs12206094-T	5´- CAC TTG CTT TGC CAC CAA GAA TTG C-3´<br>5´- CAC TTG CTT TGC TAC CAA GAA TTG C-3´
**C. Sequences of primers used in endpoint-PCR (analysis of tissue-specific expression patterns)**	
FOXO3A<br>(NM_001455.3)	Forward: GGCAAAGCAGACCCTCAAAC<br>Reverse: GCTCGAACCCGGAATGGTAA<br>Amplicon: 504 bp
SRF<br>(NM_003131.2)	Forward: CTCAACTCGCCAGACTCTCC<br>Reverse: CCGGCTTCAGTGTGTCCTTG<br>Amplicon: 142 bp
PDX<br>(NM_000209.3)	Forward: CAGTGGGCAGGCGGC<br>Reverse: TCAACATGACAGCCAGCTCC<br>Amplicon: 154 bp
STAT5A<br>(NM_001288718.1)	Forward: GTCCTGAAGACCCAGACCAA<br>Reverse: TCAGGATCTCACCACTGCA<br>Amplicon: 172
CTCF<br>(NM_006565.3)	Forward: TCTGACAGTGAAAATGCTGA<br>Reverse: TCTGGTCTTCAACCTGAATG<br>Amplicon: 202
GAPDH<br>(NM_001289745.1)	Forward: GGCATGGCCTTCCGTGTCCC<br>Reverse: TGCCAGCCCCAGCGTCAAAG<br>Amplicon: 214

Supplementary References

1. Kent, W. J. *et al.* The human genome browser at UCSC. *Genome Res.* **12,** 996–1006 (2002).
2. Li, M. J. *et al.* dbPSHP: a database of recent positive selection across human populations. *Nucleic Acids Res.* **42,** D910–D916 (2014).
3. McGue, M. & Christensen, K. The heritability of cognitive functioning in very old adults: evidence from Danish twins aged 75 years and older. *Psychol. Aging* **16,** 272-280 (2001).
4. Bao, L., Zhou, M. & Cui, Y. CTCFBSDB: a CTCF-binding site database for characterization of vertebrate genomic insulators. *Nucleic Acids Res.* **36,** D83–D87 (2008).
5. Ziebarth, J. D., Bhattacharya, A. & Cui, Y. CTCFBSDB 2.0: a database for CTCF-binding sites and genome organization. *Nucleic Acids Res.* **41,** D188–D194 (2013).
6. Schmidt, D. *et al.* Waves of retrotransposon expansion remodel genome organization and CTCF binding in multiple mammalian lineages. *Cell* **148,** 335–348 (2012).
7. Kim, T. H. *et al.* Analysis of the vertebrate insulator protein CTCF-binding sites in the human genome. *Cell* **128,** 1231–1245 (2007).
8. Xie, X. *et al.* Systematic discovery of regulatory motifs in conserved regions of the human genome, including thousands of CTCF insulator sites. *Proc. Natl. Acad. Sci.* USA **104,** 7145–7150 (2007).
9. Schones, D. E., Smith, A. D. & Zhang, M. Q. Statistical significance of cis-regulatory modules. *BMC Bioinformatics* **8,** 19 (2007).
10. Westra, H.J. *et al.* Systematic identification of trans eQTLs as putative drivers of known disease associations. *Nat. Genet.* **45**, 1238–1243 (2013).
11. Mathieson, I. *et al.* Genome-wide patterns of selection in 230 ancient Eurasians. *Nature* **528,** 499–503 (2015).
12. Pollard, K.S., Hubisz, M.J., Rosenbloom, K.R. & Siepel, A. Detection of nonneutral substitution rates on mammalian phylogenies. *Genome Res.* **20**, 110–121 (2010).
13. Grantham, R. Amino acid difference formula to help explain protein evolution. *Science* **185**, 862-864 (1974).
14. Adzhubei, I.A. *et al.* A method and server for predicting damaging missense mutations. *Nat. Methods* **7**, 248–249 (2010).
15. Calabrese, R., Capriotti, E., Fariselli, P., Martelli, P.L. & Casadio, R. Functional annotations improve the predictive score of human disease-related mutations in

proteins. *Hum Mutat* **30**, 1237–1244 (2009).
16. Li, B. *et al.* Automated inference of molecular mechanisms of disease from amino acid substitutions. *Bioinformatics* **25**, 2744–2750 (2009).
17. Ng, P.C. & Henikoff, S. Predicting deleterious amino acid substitutions. *Genome Res.* **11**, 863–874 (2001).
18. Schwarz, J.M., Cooper, D.N., Schuelke, M. & Seelow, D. MutationTaster2: mutation prediction for the deep-sequencing age. *Nat. Meth.* **11**, 361–362 (2014).
19. Reva, B., Antipin, Y. & Sander, C. Predicting the functional impact of protein mutations: application to cancer genomics. *Nucleic Acids Res.* **39**, e118 (2011).
20. Shihab, H.A. *et al.* Predicting the functional, molecular, and phenotypic consequences of amino acid substitutions using hidden Markov models. *Hum. Mutat.* **34**, 57–65 (2013).
21. Knecht, C. *et al.* IMHOTEP - a composite score integrating popular tools for predicting the functional consequences of non-synonymous sequence variants. *Nucleic Acids Res.* pii:gkw 886 (2016).
