# Supplementary material for: Identification and characterization of two functional variants in the human longevity gene FOXO3
Source: Nat Commun. 2017 Dec 12;8:2063. doi: 10.1038/s41467-017-02183-y (PMC5727304; doi:10.1038/s41467-017-02183-y)
Supplement: Supplementary file 2 — Description of Additional Supplementary Files [file 41467_2017_2183_MOESM2_ESM.pdf]

Description of Additional Supplementary Files

File Name: Supplementary Data 1
Description: List of 1,106 SNVs detected in the FOXO3 gene region.

File Name: Supplementary Data 2
Description: Association statistics for the 205 successfully genotyped SNVs in the German whole study population and centenarian subpopulation.

File Name: Supplementary Data 3
Description: Primers used in FOXO3 targeted enrichment.
